# Not seeing the grass for the trees: Timber plantations and agriculture shrink tropical montane grassland by two-thirds over four decades in the Palani Hills, a Western Ghats Sky Island

**DOI:** 10.1371/journal.pone.0190003

**Published:** 2018-01-10

**Authors:** M. Arasumani, Danish Khan, Arundhati Das, Ian Lockwood, Robert Stewart, Ravi A. Kiran, M. Muthukumar, Milind Bunyan, V. V. Robin

**Affiliations:** 1 INTACH-Kodaikanal, Melati, Wilbet house, Kodaikanal, India; 2 The Gandhigram Rural Institute, Gandhigram, Dindigul, Tamil Nadu, India; 3 Indian Institute of Science Education and Research Tirupati (IISER-Tirupati), Mangalam, Tirupati, India; 4 Arundhati Das, Samvriddhi Gardenia, Byatarayanapura, Bangalore, India; 5 Overseas School of Colombo, Sri Lanka; 6 Vattakanal Conservation Trust, Vattakanal, Kodaikanal, India; 7 Botanical Survey of India, Coimbatore, Tamil Nadu, India; 8 Ashoka Trust for in Research on Ecology and the Environment, Srirampura, Bangalore, India; Centre for Cellular and Molecular Biology, INDIA

## Abstract

Tropical montane habitats, grasslands, in particular, merit urgent conservation attention owing to the disproportionate levels of endemic biodiversity they harbour, the ecosystem services they provide, and the fact that they are among the most threatened habitats globally. The Shola Sky Islands in the Western Ghats host a matrix of native forest-grassland matrix that has been planted over the last century, with exotic timber plantations. The popular discourse on the landscape change is that mainly forests have been lost to the timber plantations and recent court directives are to restore Shola forest trees. In this study, we examine spatiotemporal patterns of landscape change over the last 40 years in the Palani Hills, a significant part of the montane habitat in the Western Ghats. Using satellite imagery and field surveys, we find that 66% of native grasslands and 31% of native forests have been lost over the last 40 years. Grasslands have gone from being the dominant, most contiguous land cover to one of the rarest and most fragmented. They have been replaced by timber plantations and, to a lesser extent, expanding agriculture. We find that the spatial pattern of grassland loss to plantations differs from the loss to agriculture, likely driven by the invasion of plantation species into grasslands. We identify remnant grasslands that should be prioritised for conservation and make specific recommendations for conservation and restoration of grasslands in light of current management policy in the Palani Hills, which favours large-scale removal of plantations and emphasises the restoration of native forests.

## Introduction

Tropical montane habitats harbour great diversity but are threatened by climate change [1] and conversion to other uses [2], such as agriculture [3] and plantations [4]. These habitats do however harbour relict species [5] and disproportionately high endemism across the world [6–8] and can serve as an early warning system for the impacts of climate change [9].

Within these habitats, tropical montane grasslands have been shown to occur naturally, and exist as an alternative stable state to adjacent forests [10–12]. Despite occupying a fraction of protected habitat, montane grasslands host unique assemblages of endemic and threatened species of plants, birds, mammals and amphibians [7, 13, 14]. Grasslands also play an important role in the global carbon cycle due to the slower decomposition rates of organic materials and by allocating as much as 90% of their biomass to underground storage [15]. Finally, they serve as a water source for downstream communities in different parts of the world [7, 16, 17].

Despite their economic and ecological significance, tropical montane grasslands are often used for agriculture, livestock rearing and urbanisation [2, 7, 18, 19]. These threats are compounded by changes to fire regimes, soil fertility and the spread of invasive alien species [20–22] The flawed perception that these grasslands are ‘degraded habitats’ [23] has also resulted in the establishment of extensive plantations of exotic species for timber (ibid), biofuels and more recently, carbon sequestration at vast scales [24]. Consequently, Bond and Parr (13) characterise afforestation as ‘one of the most severe threats’ to these grasslands. Tropical montane grasslands are thus amongst the most threatened habitats today [25] and these threats are expected to amplify with anthropogenic global climate change [26].

### The Shola Sky Islands of the Western Ghats

The montane sky islands—situated within the Western Ghats-Sri Lanka global biodiversity hotspot [27]—consist of a unique natural mosaic of forest (locally called Shola) and grassland [17]. Like other tropical montane ecosystems, the sky islands of the Western Ghats have undergone significant habitat loss to plantations, agriculture and other developmental pressures [28]. These losses have exacerbated the isolation and fragmentation associated with these high-elevation mosaics and impacted gene flow in birds [29] and butterflies [30] and caused cultural divergences in birdsong [31, 32]. Grassland loss has even driven local extinction of several endemic plants *Impatiens tangachee* Bedd., *Papilionanthe subulata* (Willd.) Garay [33], *Rhododendron arboreum* Sm. ssp. *nilagiricum* (Zenker) Tagg. [34], *Strobilanthes kunthianus* (Nees) T. Anderson ex Benth. [35], and a bird—the threatened Nilgiri Pipit [36] in parts of the Palani landscape.

### Historical changes in Shola-Grasslands in the Palani Hills

The Palani Hills hold a significant portion of the sky island habitat of the Western Ghats. Historically, small populations of different human groups including the indigenous Paliyans have inhabited the lower slopes [37]. The upper plateau however, did not have a significant human population until the arrival of American missionaries and British civil servants in the early 19^th^ Century [38]. At the time this mosaic was contiguous with the High Range-Anamalais landscape to the west. The establishment of Kodaikanal as a hill station in 1845 triggered significant changes near the town including the creation of an artificial lake, and the introduction of exotic, fast-growing timber species (*Acacia mearnsii*, *Eucalyptus globulus* and *Pinus spp*.), that were introduced to meet local needs. Nonetheless, it was not until the 1960s that landscape-scale changes occurred, when the forest department began replacing montane grasslands, then categorised as ‘wasteland’ [23], with exotic timber plantations. Some plantation species–most notably *Acacia mearnsii*–are recognised invasives [39] and began invading the grassland. In 1996 there was a nationwide ban on tree felling [40], but planting activities continued. Recently, a significant part of the Palani landscape, including large tracts of plantations, was designated as the Kodaikanal Wildlife Sanctuary (KWS; 20.09.2013 G.O Ms No 143).

### Current perception of ecosystem change and restoration in the Palani Hills

Of late, there is a growing perception that these exotic timber plantations have adversely affected biodiversity and the water table, although Rangan *et al*. [41] suggests that regional climatology may be responsible for the latter. These issues have been reported across several newspapers [42] and social media platforms [43] and even resulted in a court’s interim direction to “ensure that Shola forests and tropical rain forests are restored to its original state” [44]. Despite a lack of detailed information on the extent and nature of landscape change in the Palani Hills, the state forest department has now been tasked to “annihilate wattle” (*sic*) and restore forests, assuming that only forests have been impacted by these plantations (WR1-7028-2014 dated 26-3-2014). Additionally, regional assessments of landscape change have focused on forest cover while ignoring the loss of native grasslands [45, 46] However, a preliminary assessment of the Palani Hills landscape suggested an extensive loss of grassland habitat [47].

Given current forest policy in the Palani Hills and the fact that major changes in this landscape have occurred since the 1970s, we feel that a quantitative assessment of landscape change using satellite images, with a focus on both forests and grasslands, is appropriate and necessary. Our objectives are to study landscape changes from 1973–2014, with a focus on the loss and conservation of natural habitats. Specifically, we ask:

1. What are the spatiotemporal patterns of change in grasslands, forests, plantations and agriculture in the Palani Hills?
2. What factors influence the pattern of grassland loss?
3. What is the representativeness of protected area system in this landscape given historical change?

## Methods

### Data and study area

**Fig 1:**
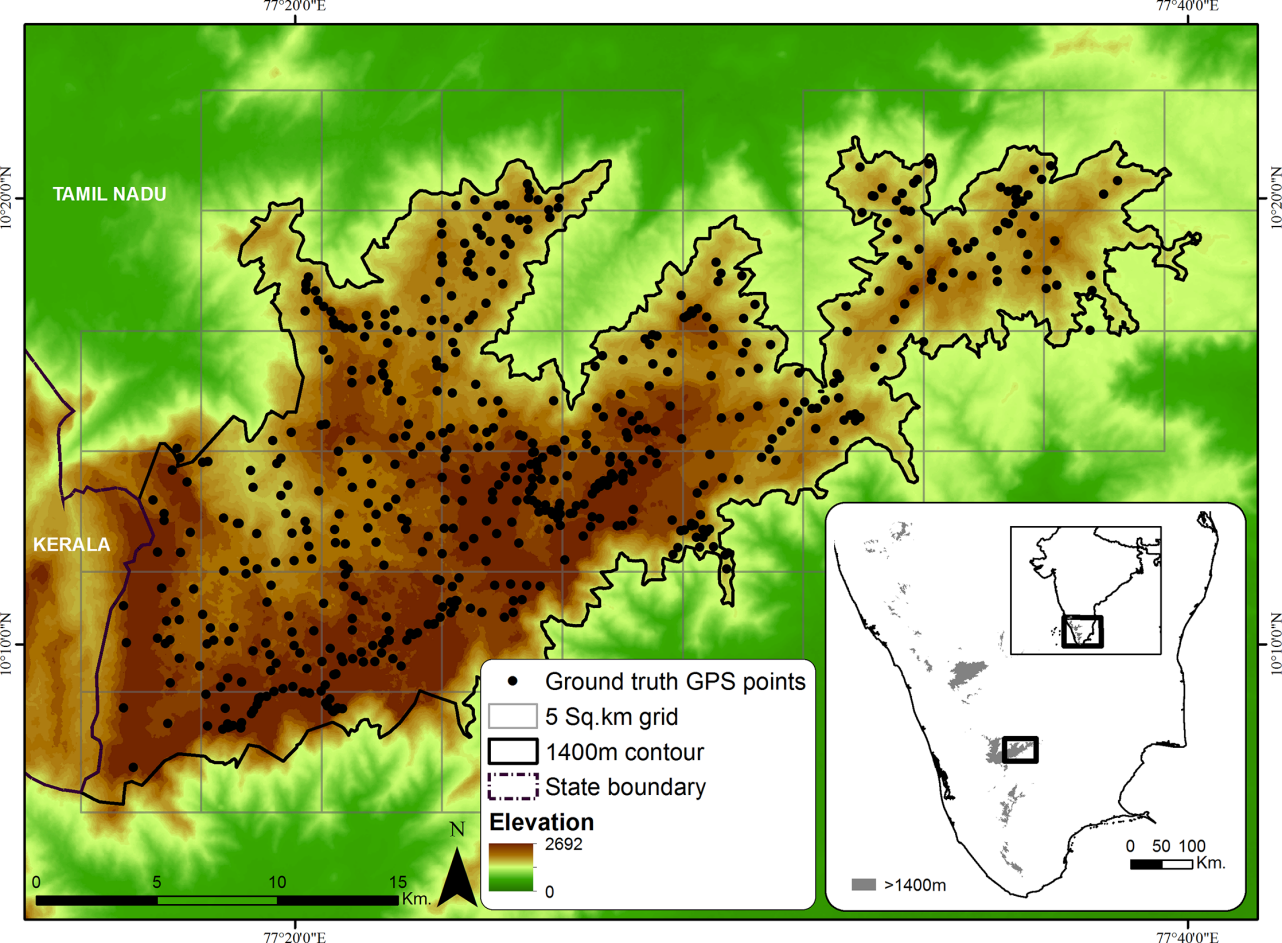
Palani Hills with ground data collected across the landscape.

We procured Landsat images from U.S. Geological Survey (https://earthexplorer.usgs.gov/) and Global Land Cover Facility (http://www.landcover.org/) for every decade (1973, 1981, 1993, 2003 & 2014) from the earliest available date (S1 Fig) for the Palani Hills landscape (S1 Table). Data were procured for the dry season when cloud cover is low and spectral differences between agriculture and grasslands are accentuated. We restricted our analysis to areas ≥ 1400m elevation; this is a conservative threshold for the occurrence of montane shola-grassland habitat [17]. On the western side, we used the state boundary to restrict our study to Tamil Nadu (Fig 1), since state governments uniquely determine forest management and this boundary has been stable for the period of the study. The study area falls in the Dindigul district, between 10° 6' to 10°20'N and 77°16' to 77°24'E.

### Image preprocessing and classification

We conducted noise reduction for the satellite remote sensing images (Landsat MSS/TM/ETM+) in the visible data. We used a self-adaptive filter method for non-periodic noise and Fast Fourier Transformation for auto removal of periodic noise [48] in ERDAS IMAGINE 2014. We then converted Digital Number (DN) of the images to radiance, and further, to retrieve only the surface reflectance, we removed the atmospheric components (e.g. water vapor, dust particles) using FLAASH in ENVI[49]. In order to bring all satellite imageries to the same geographical coordinate system for change detection studies, all imageries were georeferenced into WGS 84 datum. We then used Image-to-image registrations using Ground Control Points (GCPs) obtained from Landsat 8 OLI orthorectified data. Multi-resolution satellite imageries were resampled into 30 m resolution to reduce pseudo change detection [45]. We mapped roads and verified locations of settlements, grasslands and water bodies with Survey of India (SOI) toposheets (1:50,000) and obtained the KWS boundary from Tamil Nadu Forest Department (TNFD). For image classification and ground-truthing, we divided the study area into 5 km^2^ grids (Fig 1) and collected data on landcover. Data was gathered from 555 points between Feb 2016–Oct 2016 using Garmin (etrex Vista-H) GPS. GPS PDOP error was less than 20 m—less than the 30m resolution of the Landsat pixel. The Palani study area is spread across two LANDSAT scenes, and we were unable to obtain cloud-free data for this region in 2015 or 2016. The 2014 imagery cloud-free data was therefore the closest in time to the 2016 training samples and was used for classification. There was, however, one scene (89% of study area) that was cloud-free in 2016. We found a small (1.3%) difference between this image and the 2014 imagery (S2 Fig), and accordingly chose to use the 2014 images for the full landscape.

For the years 1973–2003, we used a hybrid classification method [45] combining supervised classification, topography and reference data (collected from the SOI toposheets), visual interpretation techniques [50] using image characteristics (e.g. tone & texture) and site-associated features to classify the landscape (S2 Table). Information on forest plantations was also collected for 1993 and 2003 from TNFD management plans. We used a supervised classification approach (Spectral Angular Mapper) to classify the landscape into six discrete landcover types–Shola Grassland, Shola Forest, Timber Plantations (includes soft and hardwood), Settlements, Agricultural and Fallow Lands, and Water bodies. We also classified the 2014 imagery with the same techniques as the 1973–2003 data, in order to assess accuracy across methods. We found that this accuracy (2014 data classified using hybrid classification method) was similar across methods.

As we were unable to classify plantation forests by species (i.e. *Acacia*, *Pinus*, and *Eucalyptus*) due to the limited resolution of the historical images, we merged them into a single class–timber plantations. Agricultural fallows and grasslands were observed to have similar reflectance. Misclassifications also occurred between bare rock and settlements and between young plantations and shola forests. These errors were addressed through reclassification in ERDAS IMAGINE (v2014) using a combination of field data and high-resolution Google Earth imagery (http://earth.google.com/). We were unable to accurately demarcate settlements from agriculture in our supervised classification. We addressed this by digitising agriculture and settlements from Google Earth imagery for 2014 (http://earth.google.com/). For earlier dates, we used SOI toposheets (surveyed between 1972–1973) to digitise these boundaries along with visual interpretation techniques.

### Accuracy assessment

We assessed the accuracy of our 2014 classification using ground truth data (150 points excluding training samples) to create an error matrix [51]. The accuracy of historical classified maps was assessed using NRSC visual interpretation techniques and toposheets [50]. To minimise errors due to differences in classification of the present (2014) and historical imagery, we also assessed the accuracy of the former (S3 Table) using NRSC’s visual interpretation techniques in addition to the assessment with ground truth data.

### Landscape change detection

We used the Landscape Change Modeler in TerrSet GIS v18.30, [52] to quantify landscape change and to calculate the area occupied by each class, after converting all the pixels to the same (30 m) resolution. We used compound interest formula to derive the (S4 Table) annual rate of change [53] in grasslands, forests and plantations.

### Spatial pattern of change

We examined change in spatial distribution of shola grassland, plantation and agriculture over the study period using metrics measuring the size distribution of patches, shape complexity, the degree of isolation and spatial dispersion of patches [54]. We also compared patterns of grassland loss to plantation versus agriculture by extracting a layer of polygons for grasslands lost to plantations, and to agriculture at each time step, and comparing their size distribution, shape complexity and spatial arrangement. These analyses were conducted in Fragstats v4 [54].

### Logistic regression

We used logistic regression models (LRMs) to assess the relationship between existing plantations and agriculture and the probability of grassland loss in the two most recent time periods (1993–2003 and 2003–2014) [55], as this was when most of the grassland loss took place (this study). Specifically, we were interested in: a) whether area under agriculture or plantation in the surrounding landscape was able to predict grassland loss to agriculture or plantation respectively, after accounting for distance to roads, settlements and topography; and b) the spatial scale at which surrounding plantations or agriculture was best able to explain grassland conversion.

As the effects of surrounding land use (plantation or agriculture) on probability of grassland conversion are not additive, we chose to model grassland loss to plantation and agriculture separately by generating Boolean images with values of ‘1’ for grassland converted to plantations and ‘0’ for grasslands remaining unchanged in 1993–2003 and in 2003–2014 (S3 and S4 Figs). We repeated this for grassland converted to agriculture in both time periods (S5 and S6 Figs).

The explanatory variables we used were: distance from major road which was normalized and log-transformed [56] (S7 and S8 Figs), proportional area under settlements in a 1050x1050m moving window (S9 and S10 Figs), proportional area under agriculture in 1993 and 2003 (in moving windows of 210x210m and 1050x1050m: S11–S14 Figs) and proportional area under plantations in 1993 and 2003 (in moving windows of 150x150m and 450x450m: S15–S20 Figs). In the case of plantations, we included a local scale (150x150m) moving window to examine effects of local invasive spread through seed rain and vegetative propagation from neighbouring plantations (Richardson and Kluge 2008). Slope variability was derived from ASTER GDEM (http://gdem.ersdac.jspacesystems.or.jp/; S21 and S22 Figs)

Two LRMs were run for each grassland conversion type (loss to agriculture, and loss to plantations) at each time step, where only the spatial scale of surrounding agriculture/plantation was varied while all other predictors were held constant(S1 R Code). We then used Akaike Information Criterion (AIC;[57]) to select the best model. A random sample of 80% of the data was used to run the LRMs, and the remaining 20% was used to validate the models. The accuracy of the LRMs predictions was assessed using the AUC_ROC_ (Area Under the Receiver Operating Characteristic Curve). This analysis was conducted in R [58] and in ArcGIS v 10.1 [59].

## Results

We were able to classify the landscape into shola forest, shola grassland, timber plantations, human settlements, agriculture and water bodies. The overall classification accuracy was 91.3%, 90.0%, 92.6%, 94.6% and 96.0% for the images from 1973, 1981, 1993, 2003, and 2014 respectively, suggesting that our classification was effective and reliable [60]. The lower accuracy for the 1973 and 1981 images was likely due to coarser spectral and spatial resolution of the Landsat MSS sensor. The Kappa coefficient was calculated to be 0.89, 0.88, 0.91, 0.94 and 0.95 for 1973, 1981, 1993, 2003 and 2014 maps respectively.

### Temporal characteristics of landscape change

**Fig 2:**
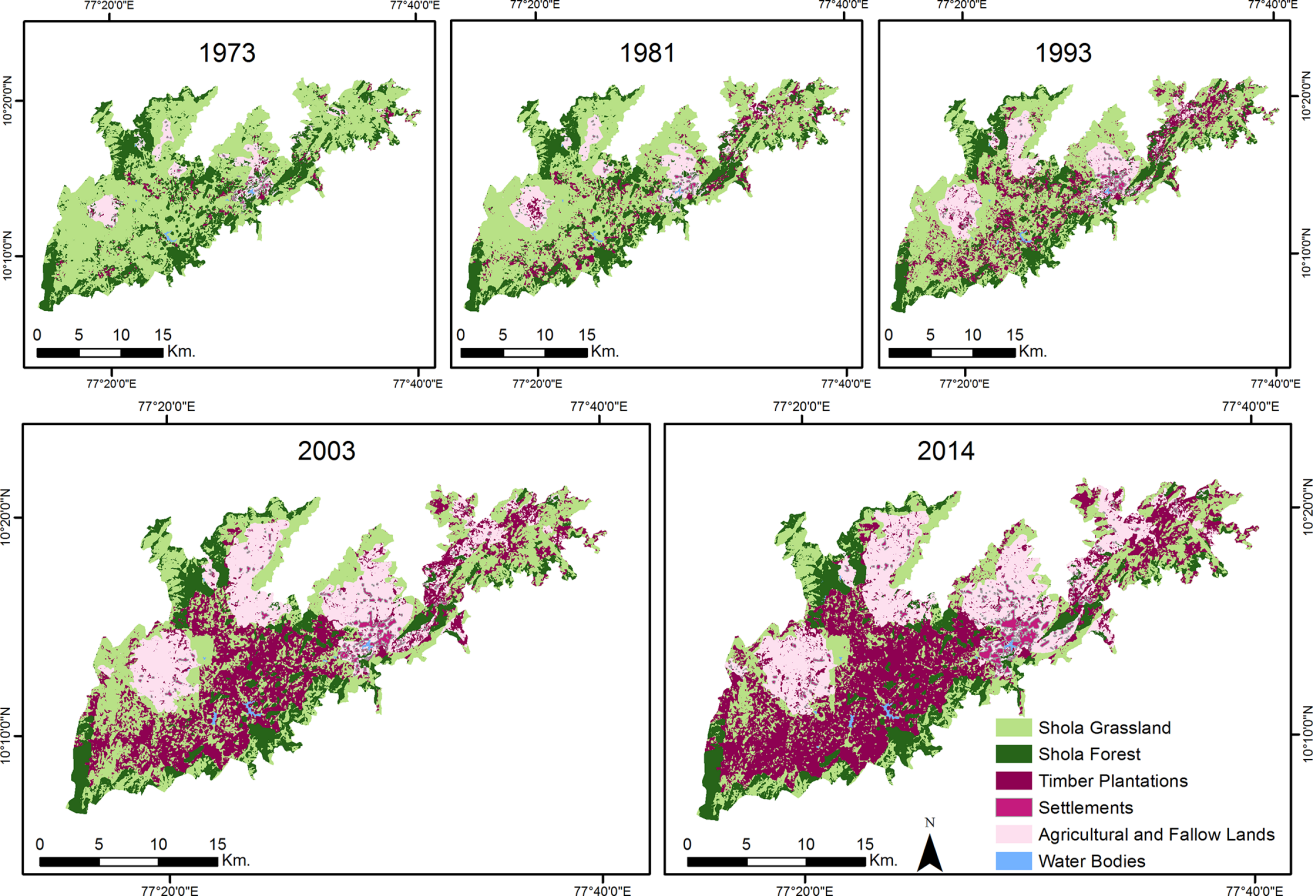
Landuse and landcover changes across the Palani Hills over four decades. GIF of this change at http://gph.is/2eBvoB2.

**Fig 3:**
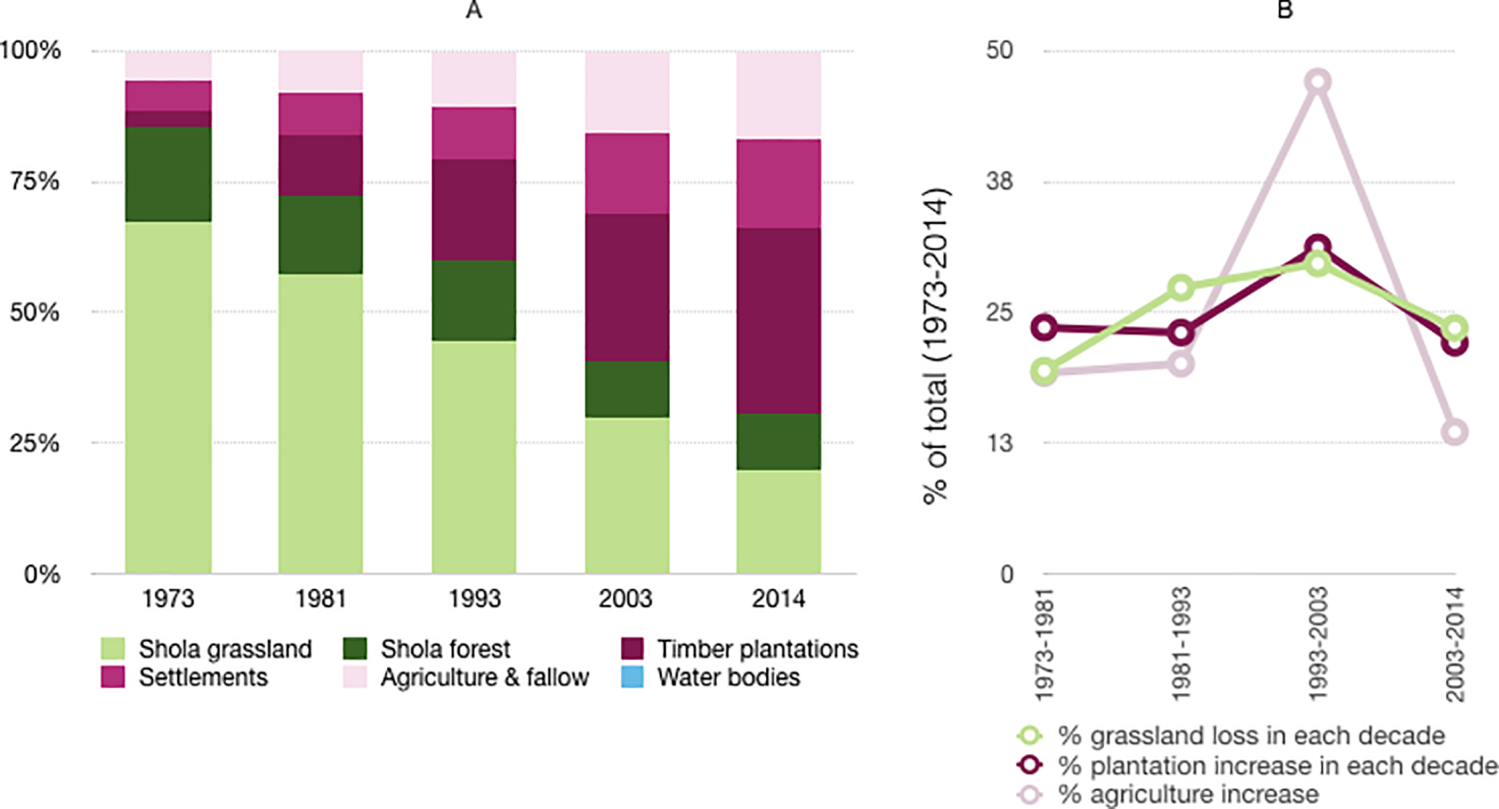
Change in landcover. (A) as expressed in the percent cover for each decade, and (B) as expressed in the decadal changes in plantation, agriculture and grassland cover as a proportion of the total change between 1973–2014 recorded for that class.

More than half (58%) of the study landscape has undergone a change from 1973–2014. This change is characterised by a disproportionate loss (249 km^2^) of Shola grasslands from 71% to 24% of the study area whereas the loss of shola forests during the same period was 33 km^2^ (Tables 1 and 2, Fig 2). The loss of native habitat was driven by a 12-fold increase in plantations, while agriculture increased from 31.1 km^2^ to 104.5 km^2^ in the same interval. Smaller increases were observed in settlements and water bodies. Between 1973–2014, area decreases (e.g. grasslands) & increases (e.g. timber plantations) were gradual (Figs 2 and 3). Nevertheless, the maximum change occurred from 1993–2014 when grassland loss and an increase in plantations and agriculture were the highest (Figs 2 and 3).

**Table 1:** Area (in sq.km.) of each classified land use type across four decades, in the Palani Hills.

LULC Class	1973	1981	1993	2003	2014
Shola Grassland	373.777	325.298	257.052	183.016	124.389
Shola Forest	99.954	87.081	89.422	65.417	66.375
Timber Plantations	18.2662	65.3104	111.4	173.727	217.9
Settlements	3.159	3.588	4.600	5.862	9.381
Agricultural and Fallow Land	31.145	45.254	60.029	94.588	104.560
Water bodies	0.726	0.491	1.044	0.998	1.033

**Table 2:** Change in area (in sq.km.) between decades, in the Palani hills, across classified land use types.

LULC Class	1973–1981	1981–1993	1993–2003	2003–2014	1973–2014
Shola Grassland	-48.479	-68.246	-74.036	-58.627	-249.388
Shola Forest	-12.873	2.340	-24.004	0.958	-33.578
Timber Plantations	47.044	46.089	62.327	44.173	199.6338
Settlements	0.428	1.012	1.261	3.519	6.221
Agricultural and Fallow Land	14.109	14.775	34.558	9.971	73.414
Water bodies	-0.234	0.552	-0.045	0.035	0.307

### Spatial pattern of landscape change

**Fig 4:**
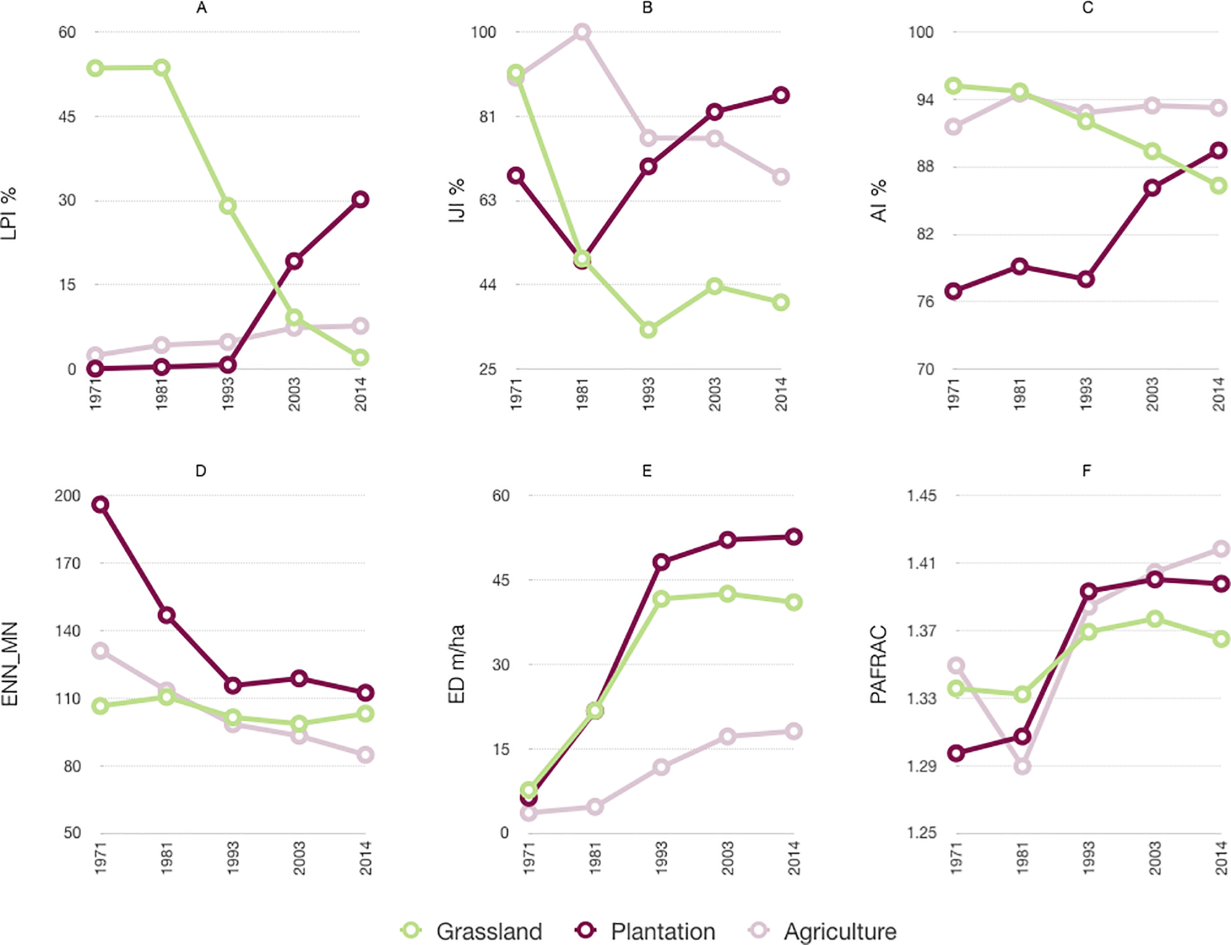
Change in the spatial characteristics of the landscape as assessed by various parameters. (A) Largest Patch Index (LPI) (B) Edge Density (ED) of the cover type (C) Mean Euclidean Nearest Neighbor Distance (ENN_MN) between patches (D) Perimeter to Area Fractal Dimension (PAFRAC) of patches (E) Aggregation Index (AI) (F) Interspersion and Juxtaposition Index (IJI) for areal extent of grasslands, plantation and agriculture between 1973 and 2014 in the Palani Hills.

The area-weighted mean (AREA_AM) grassland patch size decreased from 137.5 km^2^ ± 8.6_SD_ to 3.5 km^2^ ± 0.49_SD_, with the greatest reduction (94.7 km^2^) occurring between 1981 and 1993 (S23A Fig). In the same period, the Largest Patch Index (LPI), which measures the proportion of the landscape occupied by the largest patch of a cover type, decreased greatly for grasslands from 53% to 29% (Fig 4A). The AREA_AM plantation patch size increased from 0.15 km^2^ ± 0.05_SD_ to 95.2 km^2^ ± 3.3_SD_, with most of the increase taking place post-1993 (S23B Fig). The AREA_AM patch size for agriculture increased from 6.1 km^2^ ± 1.1_SD_ to 24 km^2^± 3.4_SD_ (S23C Fig).

#### Comparison of the spatial pattern of grassland loss to plantations with the loss to agriculture

**Fig 5:**
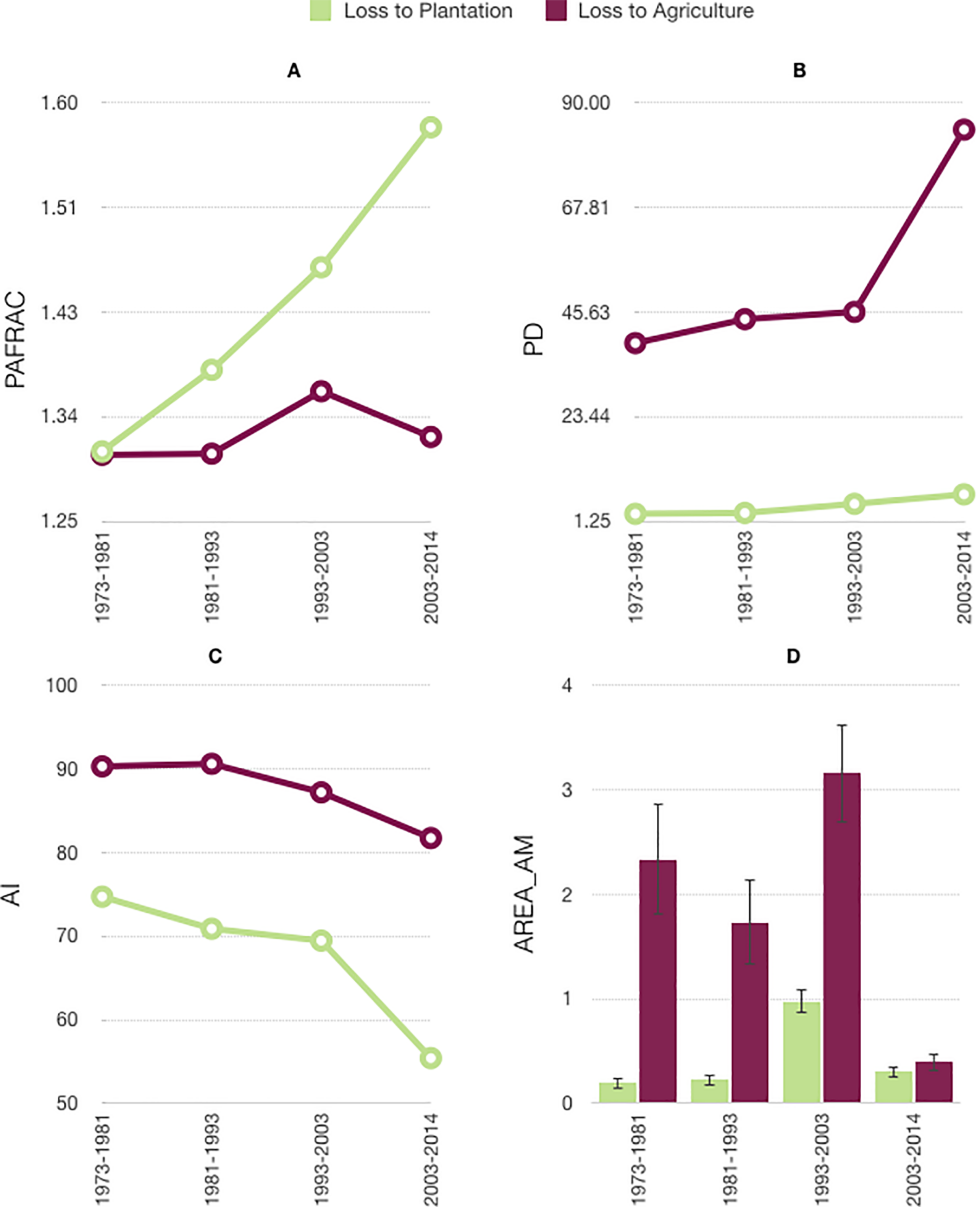
Change in spatial characteristics of grassland lost to plantation vs. grassland lost to agriculture between 1973 and 2014 in the Palani Hills. (A) Area weighted mean patch size (AREA_AM) with standard deviation (B) Perimeter-Area Fractal Dimension (PAFRAC) (C)Patch Density (PD) (D) Aggregation Index (AI).

Grassland loss to plantations (AREA_AM patch size) increased from 0.19km^2^ to 0.98 km^2^ ± 0.12_SD_ from 1973–2003, after which it decreased (Fig 5A). Although patches of grassland lost to agriculture showed higher AREA_AM values and greater levels of variation, the temporal trend was similar, with the largest patches (3.2 km^2^ ± 0.47_SD_) lost to agriculture from 1993–2003 (Fig 5B). Grassland patches lost to plantation had higher shape complexity (Perimeter to Area Fractal dimension—PAFRAC) than those lost to agriculture. Further, PAFRAC values associated with plantation-driven loss increased with time unlike the agriculture-driven loss of grassland (Fig 5B). Finally, grassland patches lost to plantations showed lower aggregation (55.4%-74.7%) than patches lost to agriculture (81.7%-90.5%, Fig 5D).

### Factors influencing grassland loss

All predictors chosen were significantly correlated to grassland loss. In the case of grasslands lost to plantations, the model with proportion area under plantation in a 150x150m window performed the best (β = 2.74, p < 0.001 in 1993–2003 and β = 4.75, p < 0.001 in 2003–2014). The proportion of plantation in the immediate neighbourhood of grasslands explained grassland loss better than the proportion of plantation at coarser scales. The AUC_ROC_ values for these models were -0.72 (1993–2003) and -0.84 (2003–2014). In the case of grasslands lost to agriculture, the area under agriculture within a 1050x1050m neighbourhood was a better predictor of grassland loss (β = 5.47, p < 0.001 in 1993–2003 and β = 7.27, p < 0.001 in 2003–2014) than area under agriculture within a 210x210m neighbourhood. The AUC_ROC_ values for these models were -0.76 (1993–2003) and -0.86 (2003–2014).

Regions with flat terrain (lower slope variability) were consistently and significantly associated with higher grassland loss. Grassland lost to agriculture was negatively related (*p* <0.001) with distance from major roads, while grasslands lost to plantation were positively related (*p* <0.05) to distance to major roads. The proportional area under settlements in a 1km^2^ neighbourhood did not show consistent correlation with grassland loss across the models.

## Discussion

We used historical and current satellite images and extensive ground data to establish the widespread loss of tropical montane habitats in the Palani Hills landscape from 1973–2014. As much as 88% of this loss occurred in Shola grasslands while the rest occurred in Shola forests, owing to conversion to timber plantations, followed by agriculture. The former increased dramatically by 1097% and the latter by 236% during this period. This loss of native habitats contradicts other studies that indicate an increase in forest cover in the Palani Hills [61, 62]. These differences could be because other studies restrict assessments to forests that ignore native grasslands or do not distinguish exotic tree plantations from native forests [45] As this study indicates, these generalisations may grossly misrepresent the extent of change to native habitats, especially with reference to Palani Hills.

### The decimation of grasslands

While most popular discourse suggests that timber plantations have replaced Shola forests, our data shows instead that in the last forty years, Shola grasslands have gone from being the dominant, most contiguous cover type to one of the rarest and most fragmented. This data supports naturalists’ careful observations [33, 63] of the threat to grasslands since the early 1980s. Today, less than 33% of the original extent of Shola grassland remains, and much of this is highly fragmented. Such loss of native grasslands has also been reported from the South American mountains [64], Qinghai-Tibet Plateau [65], and northern New Mexico in USA [66]. Although Shola forests have undergone a substantial loss in the Palani Hills, they have persisted in many locations and are even regenerating under the cover of older plantations [67]. These successional patterns have also been reported in South Africa [68–70].

#### Policy Drivers and timeline of grassland loss

Although plantations have been previously recognised as the major driver of landscape change [47], this analysis also highlights the role of settlements and agriculture. While expanding plantations drove grassland loss from 2003–2014, an increase in agriculture was primarily responsible for the loss from 1993–2003. During contact meetings with the Kodaikanal community, we learned of two events during this period that could explain our findings. The first was the settlement of Sri Lankan refugees under the Sirimavo-Shastri Pact which likely drove increases in agriculture and settlements. The second was the nationwide ban on green felling issued in 1996 [40]. This restricted Forest Department felling operations, but the establishment of new plantations continued unabated, resulting in a net increase of plantations. These two factors may have also interacted as the refugees were also used for planting operations[71]. Records of timber plantations from TNFD (S5 Table) management plans for this period roughly match our estimates for the plantation expansion in this decade.

#### Are timber plantations invading the grassland?

The spatial pattern of grassland loss to plantations is quite different from the loss to agriculture. The former has occurred in a spatially disaggregated manner with several smaller, increasingly irregularly shaped patches being lost throughout the landscape. Grassland loss to plantations, therefore, appears to be increasingly driven by natural processes, such as the invasive spread of plantation species, particularly *Acacia mearnsii*. This species is known to be a prolific seed producer and can spread vegetatively [72] In comparison, grassland loss to agriculture is characterised by relatively large, compact, spatially aggregated patches: a pattern characteristic of anthropogenic processes. This implies that grasslands in other parts will be threatened by the invading plantations unless conservation action is not taken to check this advance. Within Palani hills there are 27 species of endemic grasses among which one is near threatened [73]–all of these face significant conservation challenges.

### Current grassland extent and conservation strategy

Only half of the existing grasslands (55.05 km^2^) appear to be included in the KWS; this area is steadily dwindling by the year. The loss of grassland to date has substantially impacted the population of the endemic Nilgiri Pipit [36] and retaining existing grasslands is critical for the conservation of endemics. We identified eight grasslands along cliff edges or bordering abandoned agricultural areas that could be included in the KWS (S24 Fig). We also recommend the following conservation actions:

1. *Identify and conserve core grasslands*: Core grassland areas consist of a few to many hectares of grassland encompassing hillocks, streams, marshes and rock outcrops. These areas, even when nestled in a plantation matrix, should be protected and form the core around which grassland restoration efforts should focus.
2. *Check invasion in sparsely invaded grasslands*: These areas are often characterised by young plantations located in grasslands where grass cover is still extensive. Here, we recommend physical removal of invasive species. Forest departments often have access to significant funding through the Compensatory Afforestation (Bill passed in 2016) funds and these could be utilized for these activities. Such funds could be used for the restoration of marshes, existing grasslands and to manage the invasive plantations.
3. *Review indiscriminate removal of mature plantations*: Mature plantations often have native shola forest regenerating under them and lack native grass cover. Grassland restoration here is likely to be very resource-intensive. Conservation efforts should focus on sparsely invaded and pristine grasslands. In mature plantations, we recommend conducting experimental or controlled studies (like at Vattavada, Munnar Kerala), perhaps also examining the role of fire, and monitoring soil and moisture conditions in these areas. Moreover, removal of mature plantations could stimulate regeneration of plantation species from saturated soil seed banks [72]. Monitoring of these areas is important to assess the effectiveness of plantation removal.
4. *Contain agriculture*: Our field surveys indicate that paddy cultivation has been discontinued in some marshes. Given the critical role of these marshes in regulating local hydrology, efforts should be made to contain agriculture to the current extent and restore these marshes using a community-led conservation effort.

Our analysis was limited by the spatial and spectral resolution of LANDSAT imagery. Hence we were unable to differentiate timber plantations by species or identify sparsely invaded grasslands. Future work should use high-resolution imagery to identify grasslands for restoration. Despite these limitations, we believe this study provides the first quantitative assessment of change in this landscape. We provide unambiguous evidence for the loss of montane grassland to plantations and agriculture and indirect evidence for ongoing invasion by plantation species. Given the considerably greater threat to shola grasslands compared to shola forests from plantations and climate change, we recommend prioritisation of efforts to conserve and restore grasslands. We recommend that the need of the hour is a nuanced restoration model, developed in collaboration with forest managers and other stakeholders. We also caution that the large-scale removal of mature plantations as currently implemented may be counterproductive, causing further ecological damage. Conserving remnant grasslands and stemming the advance of invading plantations offers the best chance to secure the future of grasslands in this landscape.

## Supporting information

S1 Table Satellite imageries details. (PDF)

S2 Table NRSC image interpretation techniques. (PDF)

S3 Table 2014 Landuse and landcover accuracy assessment using Ground control points and NRSC interpretation techniques. (PDF)

S4 Table Annual change in grasslands, forests and plantations in the Palani Hills. (PDF)

S5 Table TNFD plantation statistics in comparison with this study’s remote-sensing–based plantation statistics. (PDF)

S1 Fig False color composite images—Landsat imageries of the Palani Hills study area. (PDF)

S2 Fig Overall landscape change from 2014–2016 in the Palani Hills. (PDF)

S3 Fig Dependent variable used for calibration of LRM– 1993–2003 grassland loss due to plantation. (PDF)

S4 Fig Dependent variable used for calibration of LRM– 2003–2014 grassland loss due to plantation. (PDF)

S5 Fig Dependent variable used for calibration of LRM– 1993–2003 grassland loss due to agriculture. (PDF)

S6 Fig Dependent variable used for calibration of LRM– 2003–2014 grassland loss due to agriculture. (PDF)

S7 Fig Independent variable used for calibration of LRM –1993 Distance from road. (PDF)

S8 Fig Independent variable used for calibration of LRM –2003 Distance from road. (PDF)

S9 Fig Independent variable used for calibration of LRM– 1993 settlements 35 cells moving window. (PDF)

S10 Fig Independent variable used for calibration of LRM– 2003 settlements 35 cells moving window. (PDF)

S11 Fig Independent variable used for calibration of LRM– 1993 agriculture 7 cells window. (PDF)

S12 Fig Independent variable used for calibration of LRM– 2003 agriculture 7 cells window. (PDF)

S13 Fig Independent variable used for calibration of LRM –1993 agriculture 35 cells window. (PDF)

S14 Fig Independent variable used for calibration of LRM –2003 agriculture 35 cells window. (PDF)

S15 Fig Independent variable used for calibration of LRM– 1993 plantation 5 cells window. (PDF)

S16 Fig Independent variable used for calibration of LRM– 2003 plantation 5 cells window. (PDF)

S17 Fig Independent variable used for calibration of LRM– 1993 plantation 7 cells window. (PDF)

S18 Fig Independent variable used for calibration of LRM– 2003 plantation 7 cells window. (PDF)

S19 Fig Independent variable used for calibration of LRM– 1993 plantation 15 cells window. (PDF)

S20 Fig Independent variable used for calibration of LRM– 2003 plantation 15 cells window. (PDF)

S21 Fig Independent variable used for calibration of LRM– 1993 slope variability. (PDF)

S22 Fig Independent variable used for calibration of LRM– 2003 slope variability. (PDF)

S23 Fig (a) Change in Area-weighted mean patch size (AREA_AM) for grasslands between 1973–2014 (b) Change in AREA_AM for plantations between 1973–2014 (c) Change in AREA_AM for agriculture between 1973–2014.
(PDF)

S24 Fig Grasslands inside and outside of product area. (PDF)

S1 R Code logistic regression modeling. (PDF)

## References

[1] Still CJ, Foster PN, Schneider SH. Simulating the effects of climate change on tropical montane cloud forests. Nature. 1999;398(6728):608–10.

[2] Chazdon RL. Tropical forest recovery: legacies of human impact and natural disturbances. Perspectives in Plant Ecology, evolution and systematics. 2003;6(1):51–71.

[3] Ramírez-Marcial N, González-Espinosa M, Williams-Linera G. Anthropogenic disturbance and tree diversity in montane rain forests in Chiapas, Mexico. Forest ecology and management. 2001;154(1):311–26.

[4] Lamb D. Large‐scale ecological restoration of degraded tropical forest lands: the potential role of timber plantations. Restoration ecology. 1998;6(3):271–9.

[5] Fjeldså J, Lambin E, Mertens B. Correlation between endemism and local ecoclimatic stability documented by comparing Andean bird distributions and remotely sensed land surface data. Ecography. 1999;22(1):63–78.

[6] Balete DS, Heaney LR. Density, biomass, and movement estimates for murid rodents in mossy forest on Mount Isarog, southern Luzon, Philippines. Ecotropica. 1997;3:91–100.

[7] Burgess N, Butynski T, Cordeiro N, Doggart N, Fjeldså J, Howell K, et al The biological importance of the Eastern Arc Mountains of Tanzania and Kenya. Biological conservation. 2007;134(2):209–31.

[8] Leo M. The importance of tropical montane cloud forest for preserving vertebrate endemism in Peru: the Rio Abiseo National Park as a case study. Tropical montane cloud forests: Springer; 1995 p. 198–211.

[9] Martin PH, Bellingham PJ. Towards integrated ecological research in tropical montane cloud forests. Journal of Tropical Ecology. 2016;32(5):345–54.

[10] Bond WJ, Silander JA Jr, Ranaivonasy J, Ratsirarson J. The antiquity of Madagascar’s grasslands and the rise of C4 grassy biomes. Journal of Biogeography. 2008;35(10):1743–58.

[11] Sukumar R, Suresh H, Ramesh R. Climate change and its impact on tropical montane ecosystems in southern India. Journal of Biogeography. 1995:533–6.

[12] Henderson JV, Squires TL, Storeygard A, Weil DN. The Global Spatial Distribution of Economic Activity: Nature, History, and the Role of Trade. National Bureau of Economic Research, 2016. 10.1093/qje/qjx030 PMC6889963 31798191

[13] Bond WJ, Parr CL. Beyond the forest edge: ecology, diversity and conservation of the grassy biomes. Biological Conservation. 2010;143(10):2395–404.

[14] Jansen R, Makaka L, Little IT, Dippenaar‐Schoeman A. Response of ground‐dwelling spider assemblages (Arachnida, Araneae) to montane grassland management practices in South Africa. Insect Conservation and Diversity. 2013;6(5):572–89.

[15] Gibson DJ. Grasses and grassland ecology: Oxford University Press; 2009.

[16] Peyre G. Plant diversity and vegetation of the Andean Páramo.—University of Barcelona: PhD Thesis; 2015.

[17] Robin VV, Nandini R. Shola habitats on sky islands: status of research on montane forests and grasslands in southern India. Current Science(Bangalore). 2012;103(12):1427–37.

[18] Adler PB, Morales JM. Influence of environmental factors and sheep grazing on an Andean grassland. Journal of Range Management. 1999:471–81.

[19] Houghton RA. The worldwide extent of land-use change. BioScience. 1994;44(5):305–13.

[20] Mack RN, Simberloff D, Mark Lonsdale W, Evans H, Clout M, Bazzaz FA. Biotic invasions: causes, epidemiology, global consequences, and control. Ecological applications. 2000;10(3):689–710.

[21] Mooney HA, Hobbs RJ. Invasive species in a changing world: CSIRO; 2000.

[22] Lejeune KD, Seastedt TR. Centaurea species: the forb that won the west. Conservation Biology. 2001;15(6):1568–74.

[23] Mudappa D, Raman TS. Rainforest restoration and wildlife conservation on private lands in the Western Ghats. Making Conservation Work. 2007:210–40.

[24] Veldman JW, Overbeck GE, Negreiros D, Mahy G, Le Stradic S, Fernandes GW, et al Where tree planting and forest expansion are bad for biodiversity and ecosystem services. BioScience. 2015;65(10):1011–8.

[25] Bond WJ. Ancient grasslands at risk. Science. 2016;351(6269):120–2. doi: 10.1126/science.aad5132 26744392 10.1126/science.aad5132

[26] Change IPoC. Climate Change 2014–Impacts, Adaptation and Vulnerability: Regional Aspects: Cambridge University Press; 2014.

[27] Myers N, Mittermeier RA, Mittermeier CG, Da Fonseca GA, Kent J. Biodiversity hotspots for conservation priorities. Nature. 2000;403(6772):853–8. doi: 10.1038/35002501 10706275 10.1038/35002501

[28] Kumar S. Survey and mapping of shola forests and grasslands in the Upper Nilgiri Plateau and assessment of human utilization of the vegetation World Wild Fund for Nature, India 1993.

[29] Robin VV, Vishnudas C, Gupta P, Ramakrishnan U, editors. Deep and wide valleys drive nested phylogeographic patterns across a montane bird community Proc R Soc B; 2015: The Royal Society. 10.1098/rspb.2015.0861 PMC4590488 26085588

[30] Sekar S, Karanth P. Flying between sky islands: the effect of naturally fragmented habitat on butterfly population structure. PloS one. 2013;8(8):e71573 doi: 10.1371/journal.pone.0071573 23936518 10.1371/journal.pone.0071573 PMC3731288

[31] Purushotham CB, Robin VV. Sky island bird populations isolated by ancient genetic barriers are characterized by different song traits than those isolated by recent deforestation. Ecology and Evolution. 2016;6(20):7334–43. doi: 10.1002/ece3.2475 28725401 10.1002/ece3.2475 PMC5513277

[32] Robin VV, Katti M, Purushotham C, Sancheti A, Sinha A. Singing in the sky: song variation in an endemic bird on the sky islands of southern India. Animal Behaviour. 2011;82:513–20.

[33] Matthew KM. A report on the conservation status of south Indian plants. Biodiversity and Conservation. 1999;8(6):779–96.

[34] Giriraj A, Irfan-Ullah M, Ramesh B, Karunakaran P, Jentsch A, Murthy M. Mapping the potential distribution of Rhododendron arboreum Sm. ssp. nilagiricum (Zenker) Tagg (Ericaceae), an endemic plant using ecological niche modelling. Current Science. 2008:1605–12.

[35] Carine MA, Alexander JM, Scotland RW. A revision of the Strobilanthes kunthiana-group (Phlebophyllum sensu Bremekamp)(Acanthaceae). Kew Bulletin. 2004:1–25.

[36] Robin VV, Vishnudas C, Ramakrishnan U. Reassessment of the distribution and threat status of the Western Ghats endemic bird, Nilgiri Pipit Anthus nilghiriensis. Current Science. 2014;107(4):622–30.

[37] Norström C. " They call for us": strategies for securing autonomy among the Paliyans, hunter-gatherers of the Palni Hills, South India: Dept. of Social Anthropology [Socialantropologiska institutionen], Stockholms universitet; 2003.

[38] Mitchell N. The Indian Hill-Station: Kodaikanal: University of Chicago, Department of Geography; 1972.

[39] Global Invasive Species Database [Internet]. 2015. Available from: http://www.iucngisd.org/gisd/.

[40] Rosencranz A, Lélé S. Supreme Court and India's forests. Economic and political weekly. 2008:11–4.

[41] Rangan H, Kull CA, Alexander L. Forest plantations, water availability, and regional climate change: controversies surrounding Acacia mearnsii plantations in the upper Palnis Hills, southern India. Regional Environmental Change. 2010;10(2):103–17.

[42] Karnad D. Tackling the demon of eucalyptus, at last. Deccan Herald. 2014 04.10.2014.

[43] Ahrestani F. To Chop, ot Not to Chop? The issue of exoyic native trees in the western ghats. Conservation India. 2017.

[44] Kumar BA. State prepares plan to eliminate “aliens” from rain forests. The Hindu. 2013 May 13.

[45] Reddy CS, Jha C, Dadhwal V. Assessment and monitoring of long-term forest cover changes (1920–2013) in Western Ghats biodiversity hotspot. Journal of Earth System Science. 2016;125(1):103–14.

[46] Prakasam C. Land use and land cover change detection through remote sensing approach: A case study of Kodaikanal taluk, Tamil nadu. International journal of Geomatics and Geosciences. 2010;1(2):150.

[47] Lockwood I. Land Cover Changes in the Palani Hills: A Preliminary Visual Assessment. Ian Lockwood Blog. 2014;4.

[48] Chen X-L, Zhao H-M, Li P-X, Yin Z-Y. Remote sensing image-based analysis of the relationship between urban heat island and land use/cover changes. Remote sensing of environment. 2006;104(2):133–46.

[49] Guide EUs. ENVI on-line software user’s manual. ITT Visual Information Solutions. 2008.

[50] Dutta K, Reddy CS, Sharma S, Jha C. Quantification and monitoring of forest cover changes in Agasthyamalai Biosphere Reserve, Western Ghats, India (1920–2012). Current Science. 2016;110(4):508–20.

[51] Congalton RG. A review of assessing the accuracy of classifications of remotely sensed data. Remote sensing of environment. 1991;37(1):35–46.

[52] Eastman J. TerrSet: Geospatial Monitoring and Modeling Software. Clark Labs, Clark University 2015.

[53] Puyravaud J-P. Standardizing the calculation of the annual rate of deforestation. Forest Ecology and Management. 2003;177(1):593–6.

[54] McGarigal K, Cushman S, Ene E. FRAGSTATS v4: spatial pattern analysis program for categorical and continuous maps University of Massachusetts, Amherst, Massachusetts, USA goo gl/aAEbMk. 2012.

[55] McCullagh P, Nelder JA. Generalized Linear Models, no. 37 in Monograph on Statistics and Applied Probability. Chapman & Hall; 1989.

[56] Kumar R, Nandy S, Agarwal R, Kushwaha S. Forest cover dynamics analysis and prediction modeling using logistic regression model. Ecological Indicators. 2014;45:444–55.

[57] Burnham KP, Anderson DR. Multimodel inference: understanding AIC and BIC in model selection. Sociological methods & research. 2004;33(2):261–304.

[58] Team RC. R: A language and environment for statistical computing [Internet]. Vienna, Austria; 2014. 2017.

[59] ESRI A, Components M. Environmental systems research institute. California, USA. 2001.

[60] Anderson JR. A land use and land cover classification system for use with remote sensor data: US Government Printing Office; 1976.

[61] FSI. State of Forest Report 2001. Forest Survey of India, 2001.

[62] FSI. India State Forest Report, 2015. Forest Survey of India, 2015.

[63] Clewell AF, Aronson J. Ecological restoration: principles, values, and structure of an emerging profession: Island Press; 2013.

[64] Stone TA, Schlesinger P, Houghton RA, Woodwell GM. A map of the vegetation of South America based on satellite imagery. Photogrammetric Engineering and Remote Sensing. 1994;60(5):541–51.

[65] Liu L, Zhang Y, Bai W, Yan J, Ding M, Shen Z, et al Characteristics of grassland degradation and driving forces in the source region of the Yellow River from 1985 to 2000. Journal of Geographical Sciences. 2006;16(2):131–42.

[66] Coop JD, Givnish TJ. Spatial and temporal patterns of recent forest encroachment in montane grasslands of the Valles Caldera, New Mexico, USA. Journal of Biogeography. 2007;34(5):914–27.

[67] Srimathi A, Schmerbeck J, Gärtner S. Regeneration of Shola tree species under Eucalyptus plantations in Upper Palni Hills. Land-use Related Biodiversity in India. 2012:8.

[68] Geldenhuys CJ. Native forest regeneration in pine and eucalypt plantations in Northern Province, South Africa. Forest Ecology and Management. 1997;99(1):101–15.

[69] Geldenhuys C, Le Roux P, Cooper K, editors. Alien invasions in indigenous evergreen forest. The ecology and management of biological invasions in Southern Africa Proceedings of the National Synthesis Symposium on the ecology of biological invasions; 1986: Oxford University Press.

[70] Van Wyk G, Everard D, Geldenhuys C. Forest ecotone development and succession: experimental results and guidelines for forest rehabilitation and protection. Report FOR-DEA. 1995;867.

[71] Stewart R, Balear T. Restoration of southern Indian shola forests: realising community-based forest conservation in the Palni Hills of the Western Ghats. Social Change. 2003;33(2–3):115–28.

[72] Richardson DM, Kluge RL. Seed banks of invasive Australian Acacia species in South Africa: role in invasiveness and options for management. Perspectives in Plant Ecology, Evolution and Systematics. 2008;10(3):161–77.

[73] Kabeer K, Nair V. Flora of Tamil Nadu. Kolkata: Botanical Survey of India; 2009.

