# Supplementary material for: Not seeing the grass for the trees: Timber plantations and agriculture shrink tropical montane grassland by two-thirds over four decades in the Palani Hills, a Western Ghats Sky Island
Source: PLoS One. 2018 Jan 10;13(1):e0190003. doi: 10.1371/journal.pone.0190003 (PMC5761842; doi:10.1371/journal.pone.0190003)
Supplement: S1 Table — (PDF) [file pone.0190003.s001.pdf]

S1 Table. Satellite Imageries details

Satellite	Spatial Resolution	Number of Spectral Bands	Year and Month
Landsat 1 MSS	80m	4	09 February 1973
Landsat 2 MSS	80m	4	22 may 1981
Landsat-5TM	30m	7	17 February1993
Landsat 7 ETM+	30m	8	21 February 2003
Landsat 8 OLI	30m	11	11 February 2014
