# Supplementary material for: Not seeing the grass for the trees: Timber plantations and agriculture shrink tropical montane grassland by two-thirds over four decades in the Palani Hills, a Western Ghats Sky Island
Source: PLoS One. 2018 Jan 10;13(1):e0190003. doi: 10.1371/journal.pone.0190003 (PMC5761842; doi:10.1371/journal.pone.0190003)
Supplement: S2 Table — (PDF) [file pone.0190003.s002.pdf]

S2 Table. NRSC image interpretation techniques

Land use/land cover class	Image chip	Tone	Texture	Shape	Pattern	Description
Grasslands	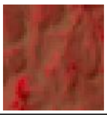	Grayish to brown Irregular	Smooth	Irregular	Scattered	Scattered Grass predominating areas
Shola	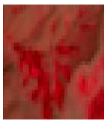	Dark red	Rough	Scattered	Rough	Evergreen forests found in between grasslands of high altitude
Plantations	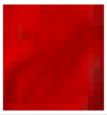	Red	Smooth	Regular	Grouped	Cultivated crop for commercial purpose
Agriculture	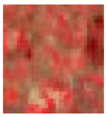	Pinkish or light green or light blue	Smooth	Regular	Smooth	Crops/current fallow lands
Settlement	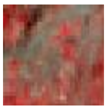	Cyan	Smooth	Regular	Scattered	Human inhabited areas
Water bodies	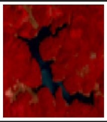	Blue or black	Smooth	Irregular	Scattered	Rivers and reservoirs
