# Supplementary material for: Not seeing the grass for the trees: Timber plantations and agriculture shrink tropical montane grassland by two-thirds over four decades in the Palani Hills, a Western Ghats Sky Island
Source: PLoS One. 2018 Jan 10;13(1):e0190003. doi: 10.1371/journal.pone.0190003 (PMC5761842; doi:10.1371/journal.pone.0190003)
Supplement: S3 Table — (PDF) [file pone.0190003.s003.pdf]

S3 Table. 2014 Landuse and landcover accuracy assessment using Ground control points and NRSC Interpretation techniques

**Class**	**2014 Based on field points**	**2014 Based on NRSC Interpretation**
Shola Forest	0.9516	0.952
Agricultural Land	0.9055	0.8049
Settlements	1	0.8583
Water bodies	0.9524	1
Grassland	1	0.856
Timber Plantations	0.9048	0.952
**Overall Kappa Statistics = 0.9520**		**0.904**
