# Supplementary material for: Not seeing the grass for the trees: Timber plantations and agriculture shrink tropical montane grassland by two-thirds over four decades in the Palani Hills, a Western Ghats Sky Island
Source: PLoS One. 2018 Jan 10;13(1):e0190003. doi: 10.1371/journal.pone.0190003 (PMC5761842; doi:10.1371/journal.pone.0190003)
Supplement: S4 Table — (PDF) [file pone.0190003.s004.pdf]

S4 Table. Annual change in grasslands, forests and plantations in the Palani Hills

LULC Class	1973 - 81	1981 - 93	1993 - 03	2003- 2014	1973 - 2014
Shola Grassland	-0.01736	-0.01962	-0.03397	-0.035105439	-0.026835
Shola forest	-0.01723	0.00221	-0.03126	0.001322064	-0.009984
Timber Plantations	0.159262	0.044498	0.044436	0.020595558	0.0604630
