# Supplementary material for: Not seeing the grass for the trees: Timber plantations and agriculture shrink tropical montane grassland by two-thirds over four decades in the Palani Hills, a Western Ghats Sky Island
Source: PLoS One. 2018 Jan 10;13(1):e0190003. doi: 10.1371/journal.pone.0190003 (PMC5761842; doi:10.1371/journal.pone.0190003)
Supplement: S5 Table — (PDF) [file pone.0190003.s005.pdf]

S5 Table. TNFD Plantation statistics in comparison with this study's remote-sensing – based plantation statistics

Range	Plantation in ha. 2014 Satellite Data result	Forest Management Plan(2010-2020)	Plantation in ha. 2003 Satellite Data result	Forest Management Plan(1996-2006)	Plantation in ha. 1993 Satellite Data result	Forest Management Plan(1984-1994)
Kodaikanal	1914.2	1796	1460.42	1940	889.47	2714
Mannavanur	3012.3	1336	2346.26	2869	1186.69	3143
Poombarai	2396.0	587	1939.79	2309	799.26	3143
Vandaravu	2815.8	1216	1695.22	2867	716.34	2495
Berijam	2609.1	798	2106.32	3254	995.66	3860
