# Supplementary figures and images for: Not seeing the grass for the trees: Timber plantations and agriculture shrink tropical montane grassland by two-thirds over four decades in the Palani Hills, a Western Ghats Sky Island
Source: PLoS One. 2018 Jan 10;13(1):e0190003. doi: 10.1371/journal.pone.0190003 (PMC5761842; doi:10.1371/journal.pone.0190003)

S1 Fig. False color composite images - Landsat imageries of the Palani Hills study area

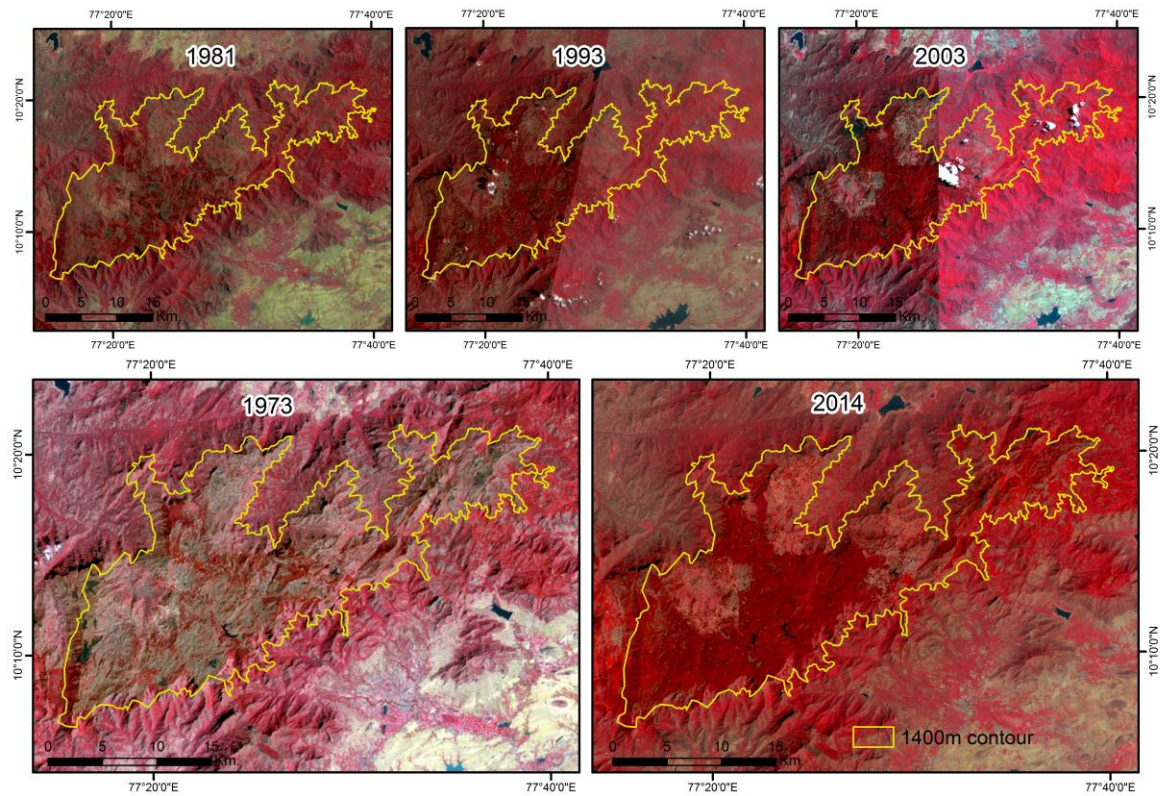

Supplement: S1 Fig — (PDF) [file pone.0190003.s006.pdf]

S2 Fig. Overall landscape change from 2014 – 2016 in the Palani Hill

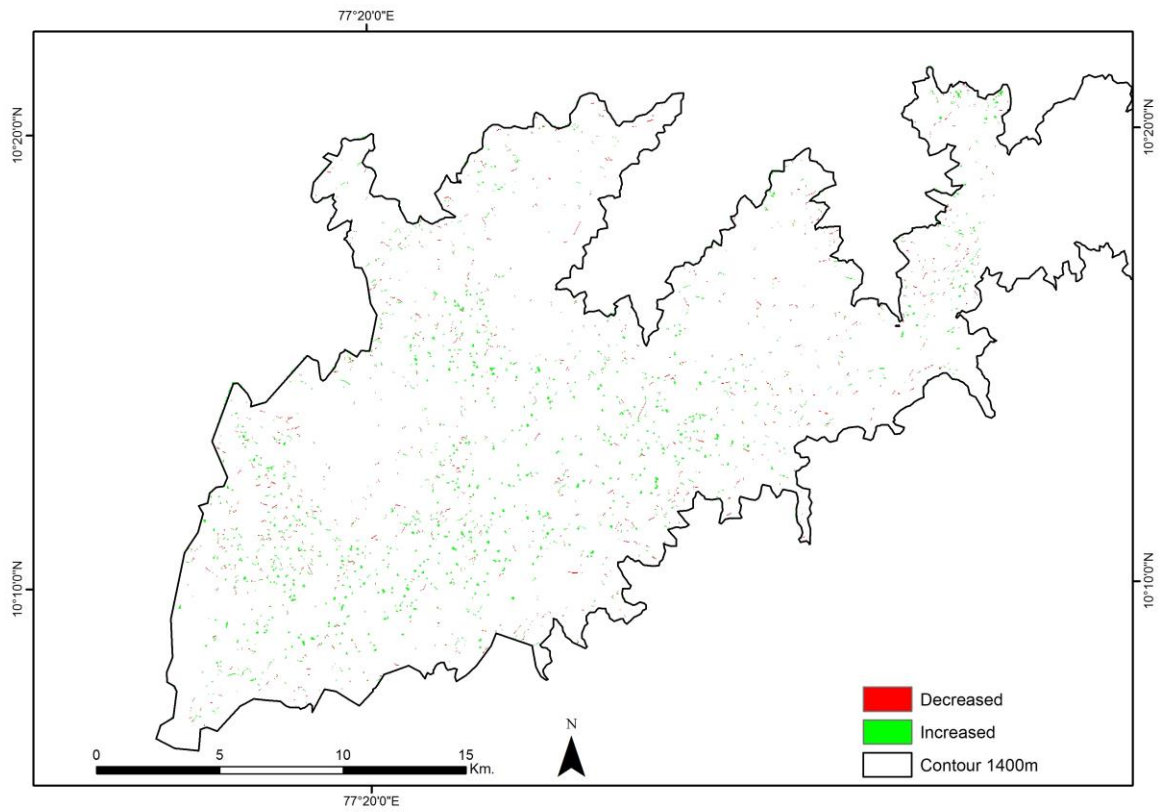

Supplement: S2 Fig — (PDF) [file pone.0190003.s007.pdf]

S3 Fig. Dependent variable used for calibration of LRM – 1993 -2003 grassland loss due to plantation

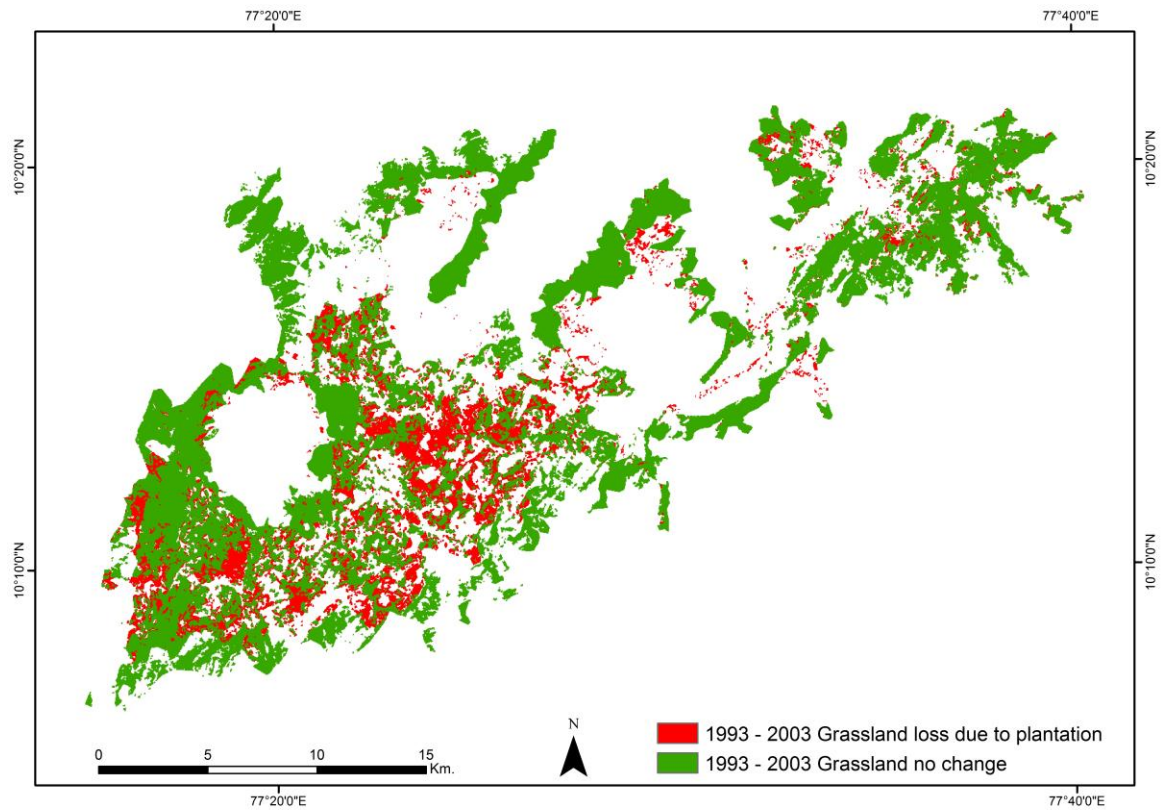

Supplement: S3 Fig — (PDF) [file pone.0190003.s008.pdf]

S4 Fig. Dependent variable used for calibration of LRM – 2003 -2014 grassland loss due to plantation

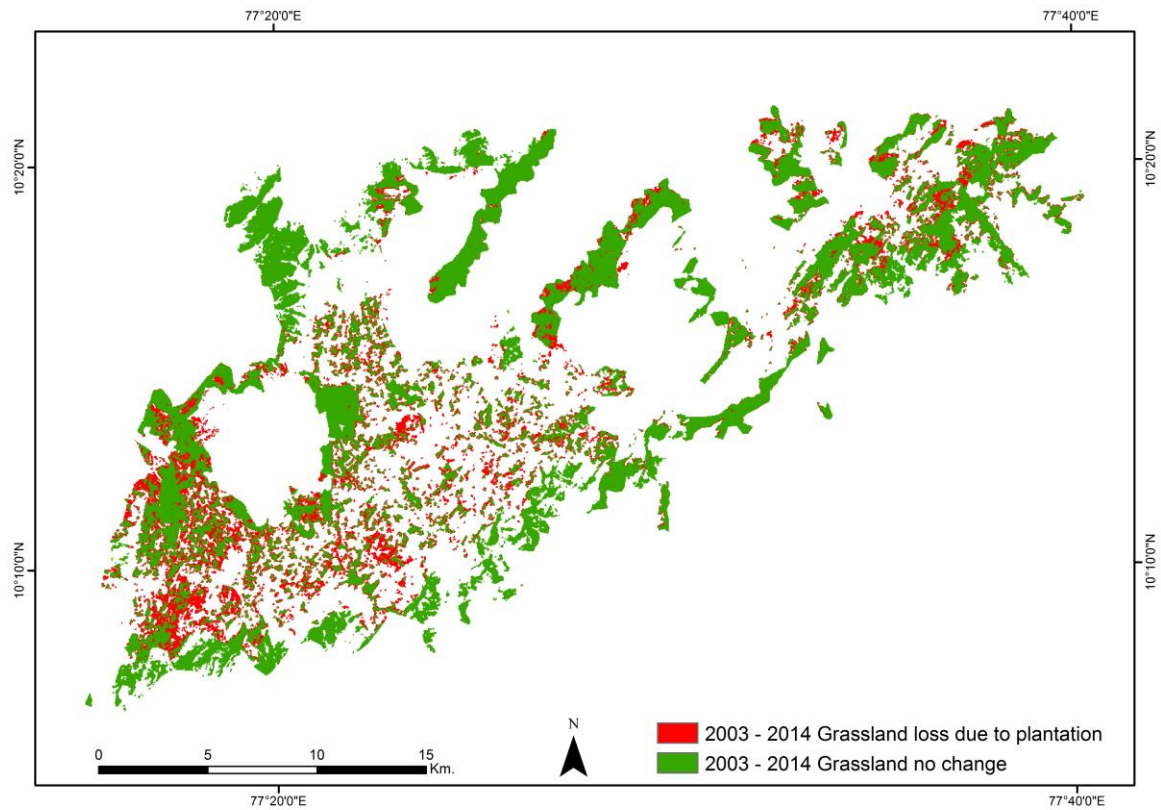

Supplement: S4 Fig — (PDF) [file pone.0190003.s009.pdf]

S5 Fig. Dependent variable used for calibration of LRM – 1993 -2003 grassland loss due to agriculture

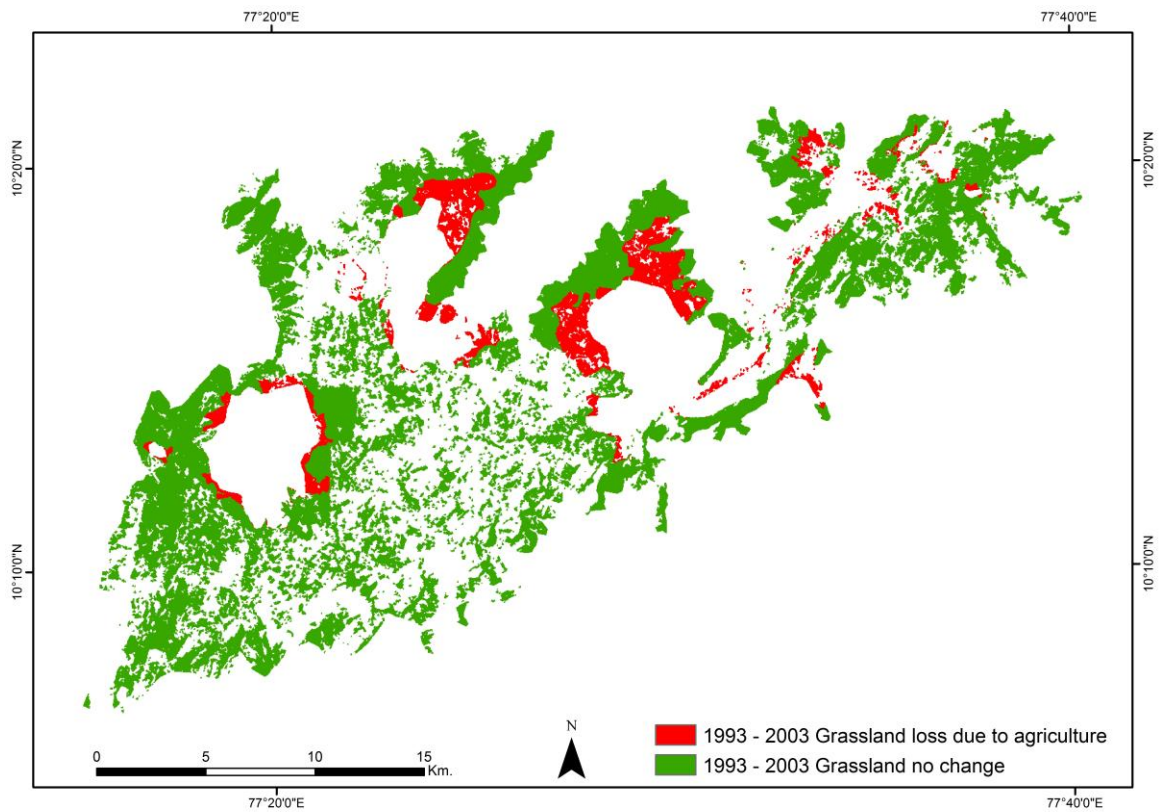

Supplement: S5 Fig — (PDF) [file pone.0190003.s010.pdf]

S6 Fig. Dependent variable used for calibration of LRM – 2003 -2014 grassland loss due to agriculture

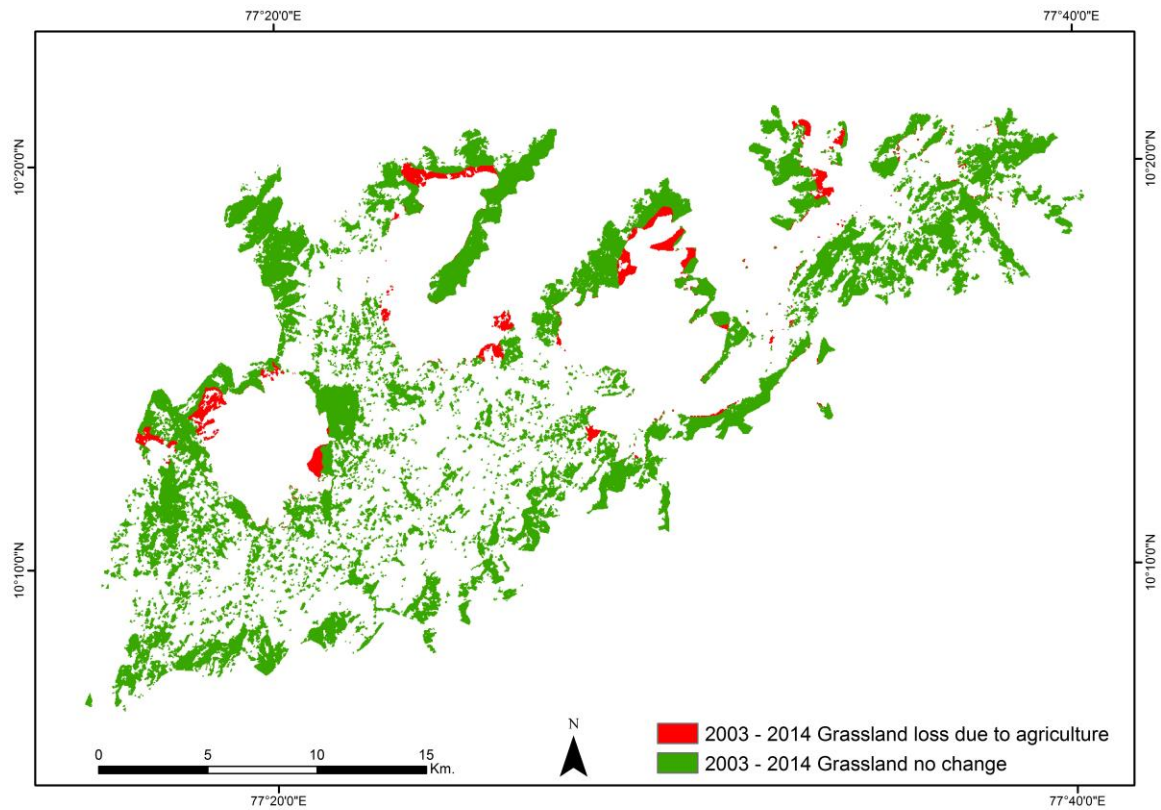

Supplement: S6 Fig — (PDF) [file pone.0190003.s011.pdf]

S7 Fig. Independent variable used for calibration of LRM –1993 Distance from road

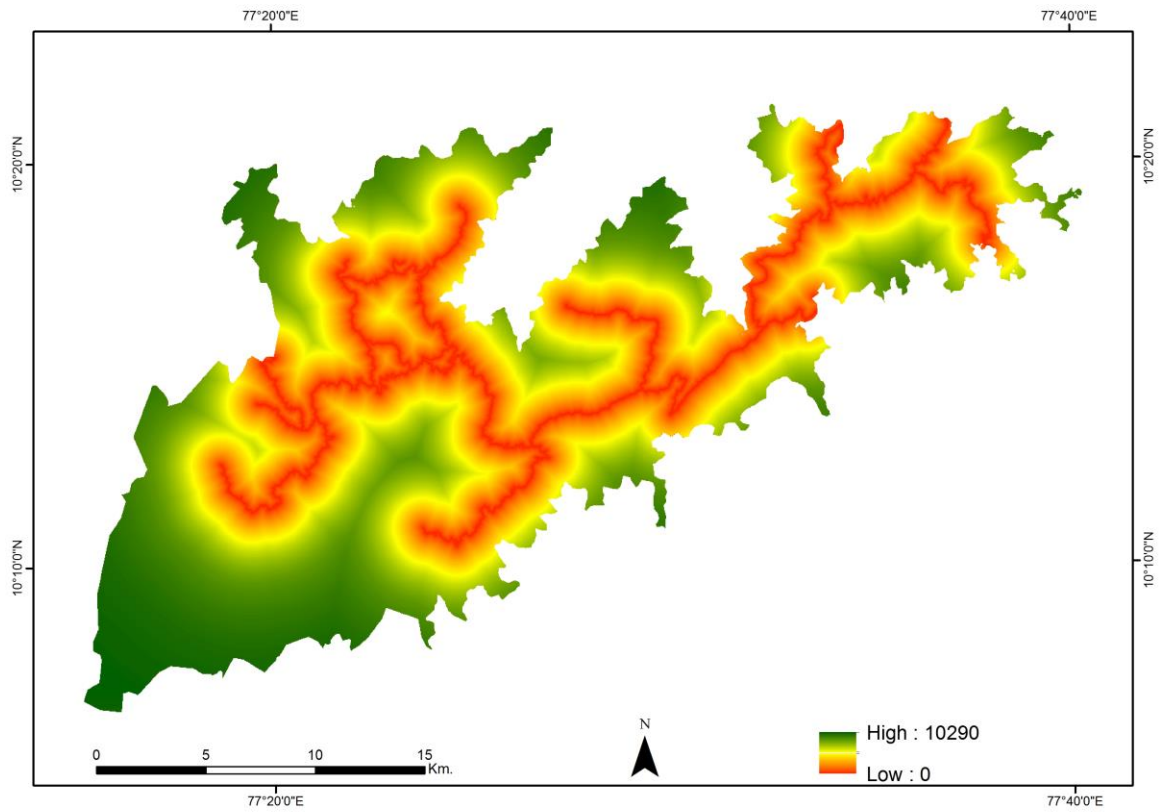

Supplement: S7 Fig — (PDF) [file pone.0190003.s012.pdf]

S8 Fig. Independent variable used for calibration of LRM –2003 Distance from road

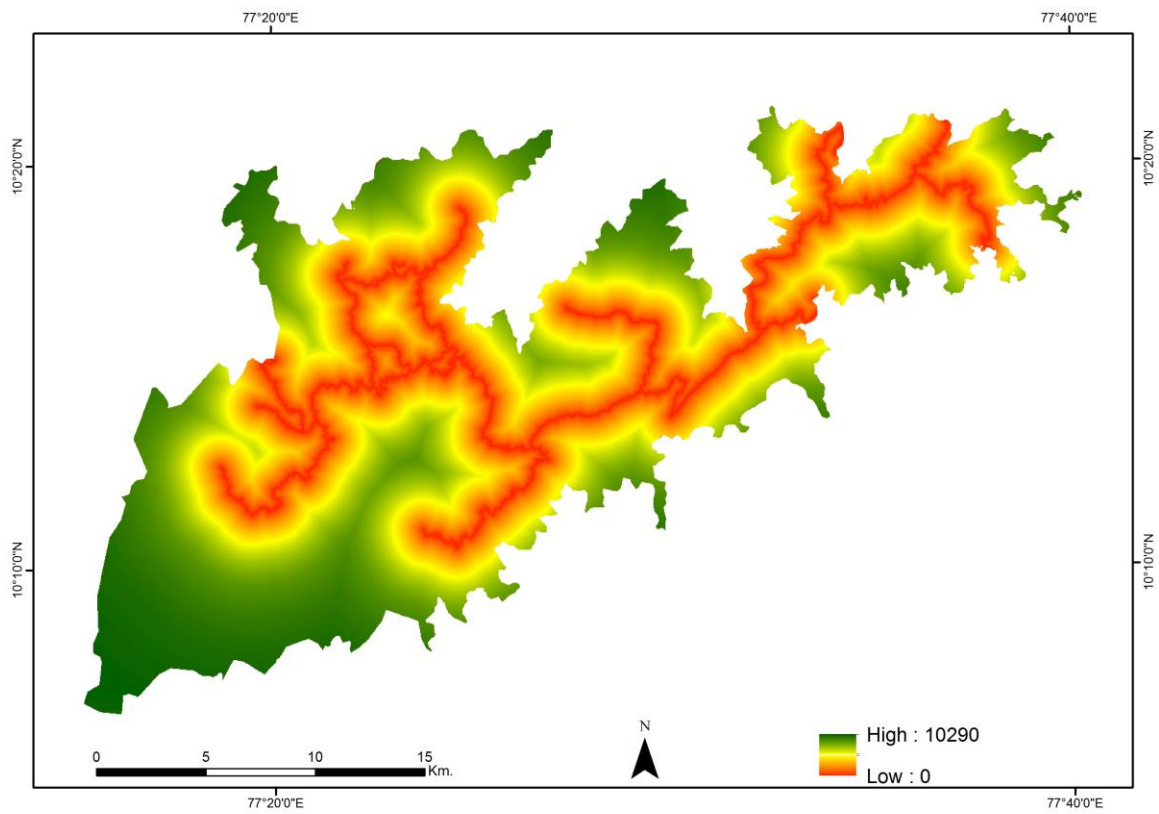

Supplement: S8 Fig — (PDF) [file pone.0190003.s013.pdf]

S9 Fig. Independent variable used for calibration of LRM – 1993 settlements 35 cells moving window

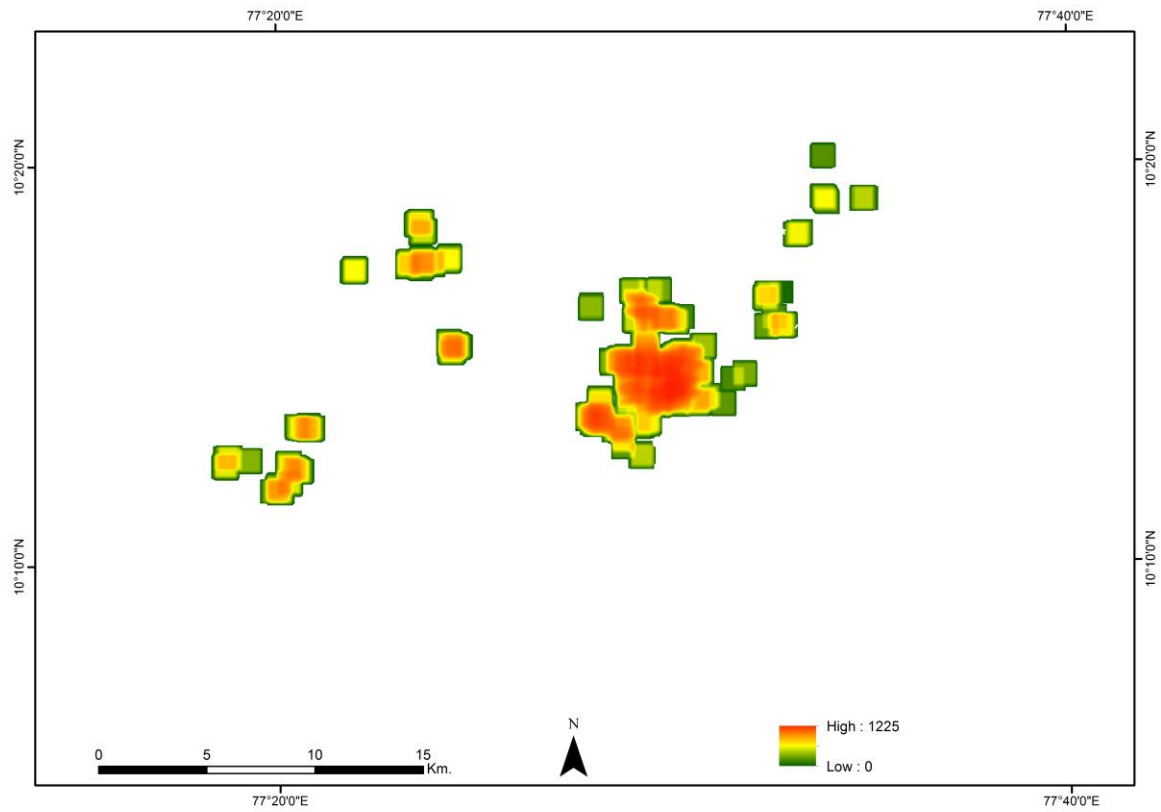

Supplement: S9 Fig — (PDF) [file pone.0190003.s014.pdf]

S10 Fig. Independent variable used for calibration of LRM – 2003 settlements 35  
cells moving window

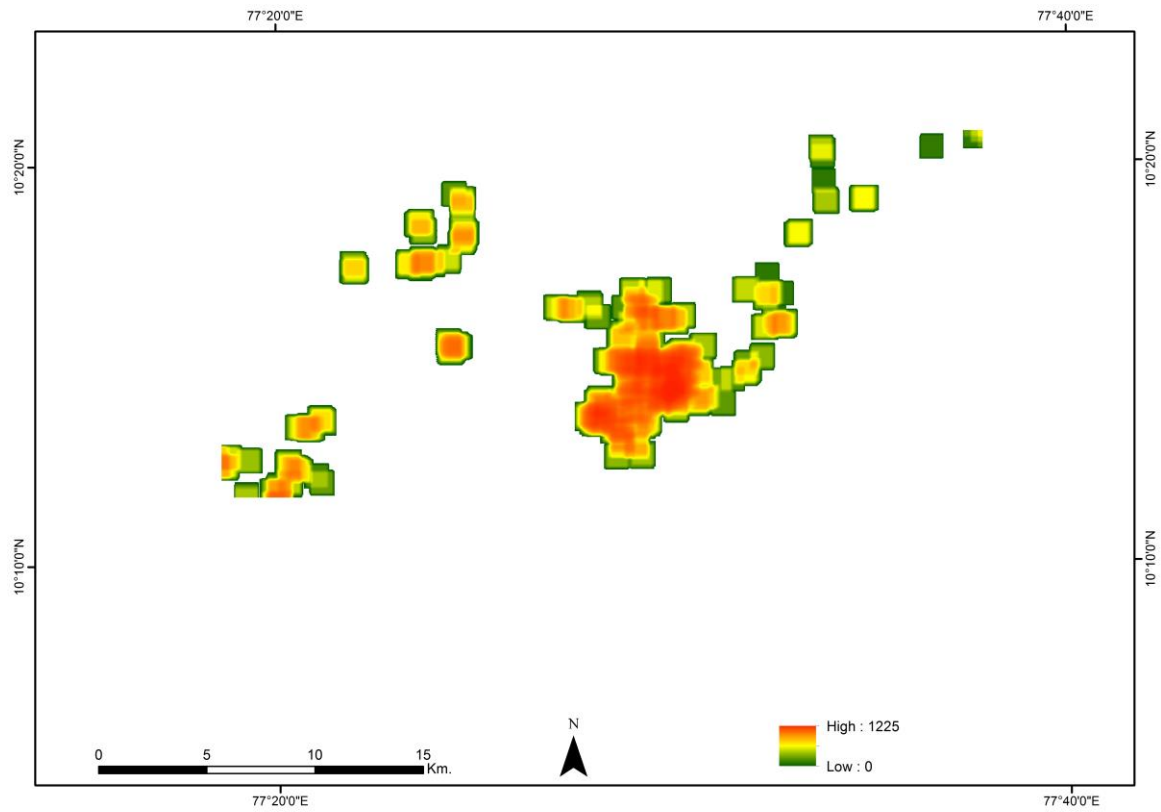

Supplement: S10 Fig — (PDF) [file pone.0190003.s015.pdf]

S11 Fig. Independent variable used for calibration of LRM – 1993 agriculture 7 cells window

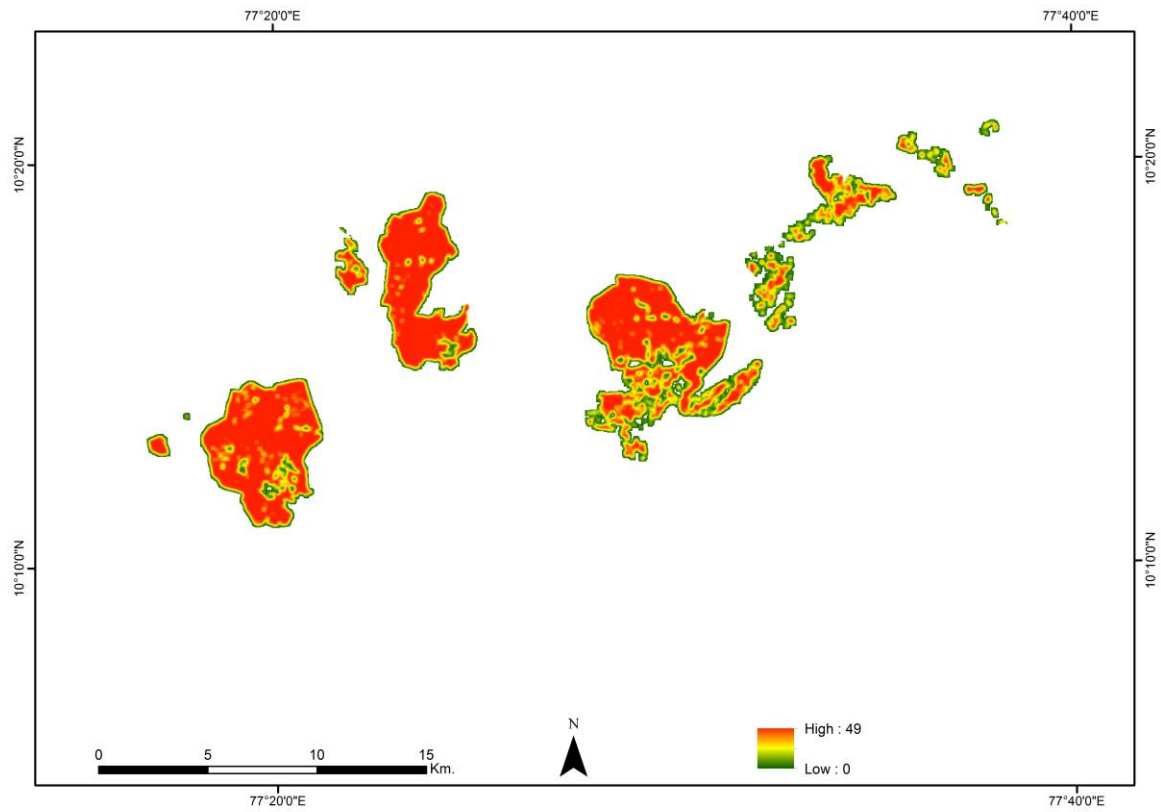

Supplement: S11 Fig — (PDF) [file pone.0190003.s016.pdf]

S12 Fig. Independent variable used for calibration of LRM – 2003 agriculture 7 cells window

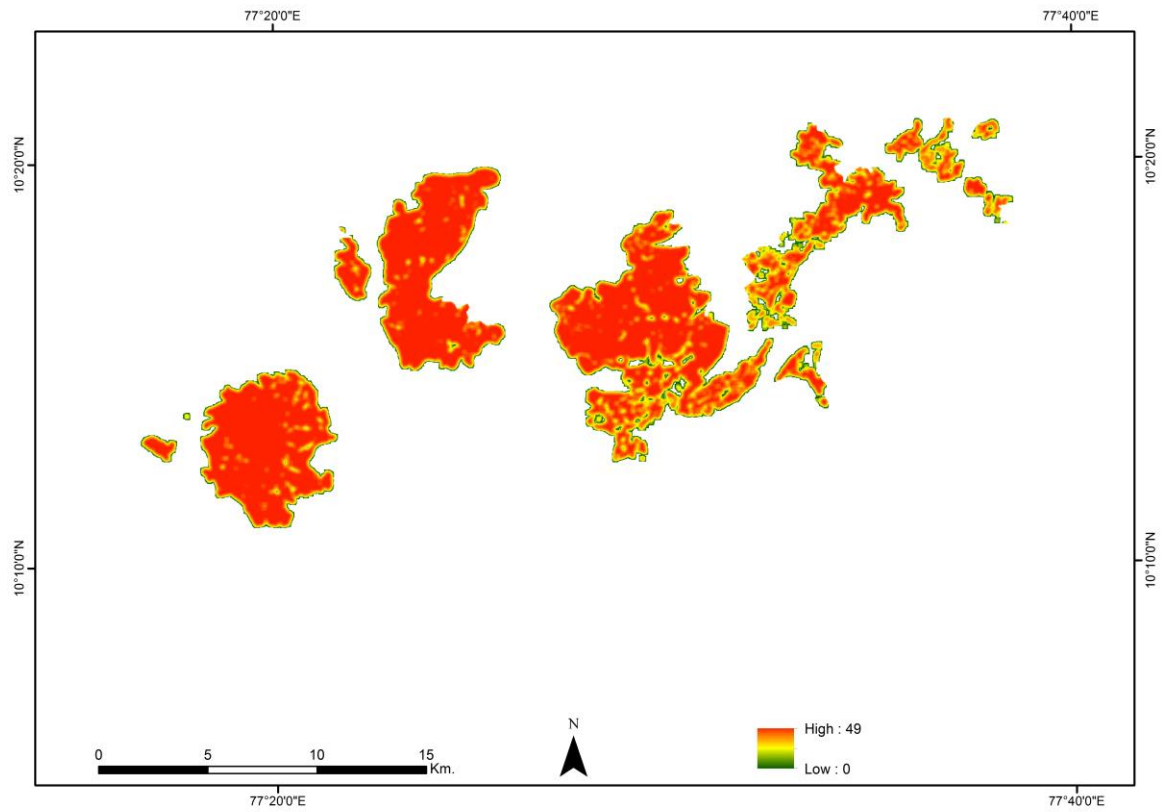

Supplement: S12 Fig — (PDF) [file pone.0190003.s017.pdf]

S13 Fig. Independent variable used for calibration of LRM –1993 agriculture 35 cells window

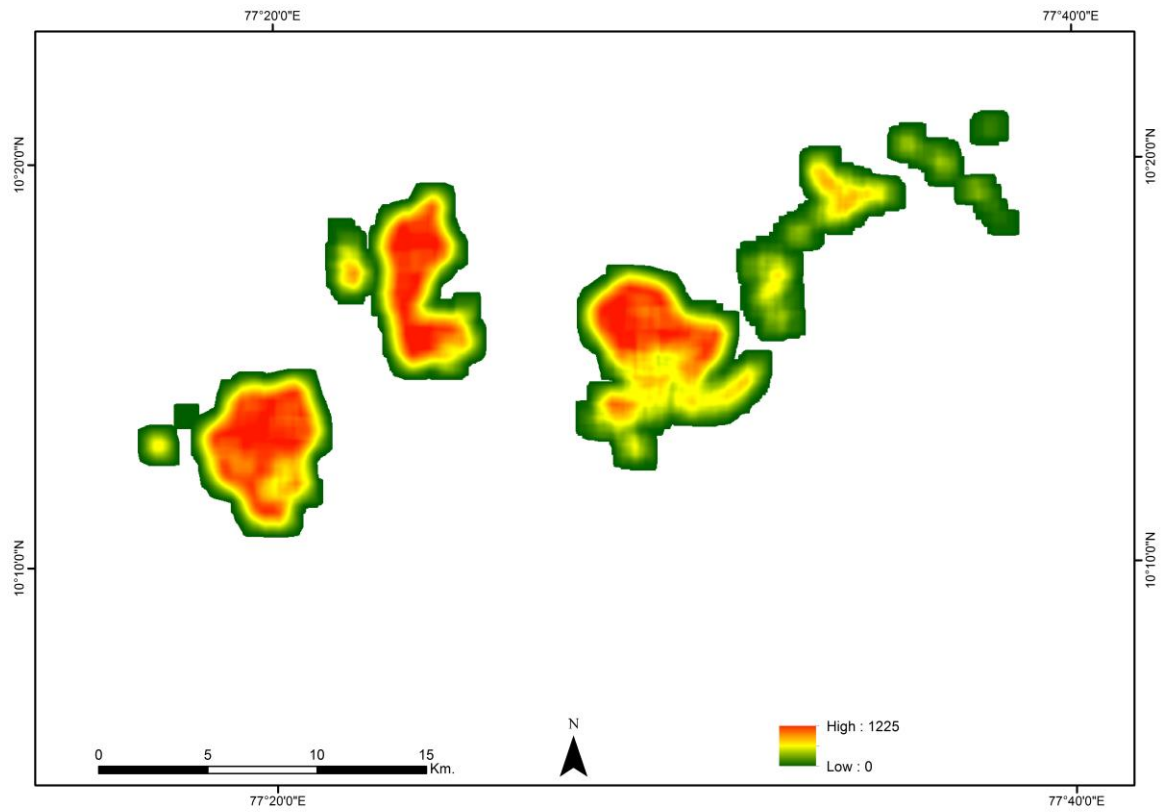

Supplement: S13 Fig — (PDF) [file pone.0190003.s018.pdf]

S14 Fig. Independent variable used for calibration of LRM –2003 agriculture 35 cells window

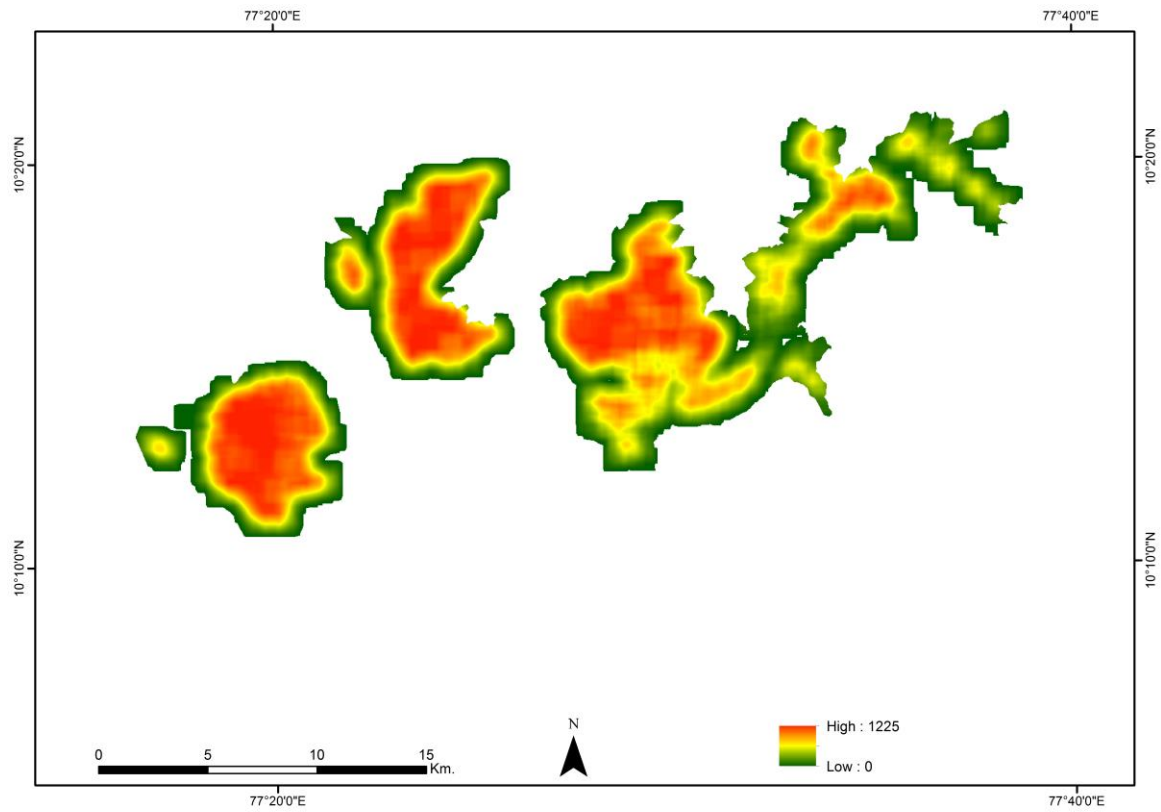

Supplement: S14 Fig — (PDF) [file pone.0190003.s019.pdf]

S15 Fig. Independent variable used for calibration of LRM – 1993 plantation 5 cells window

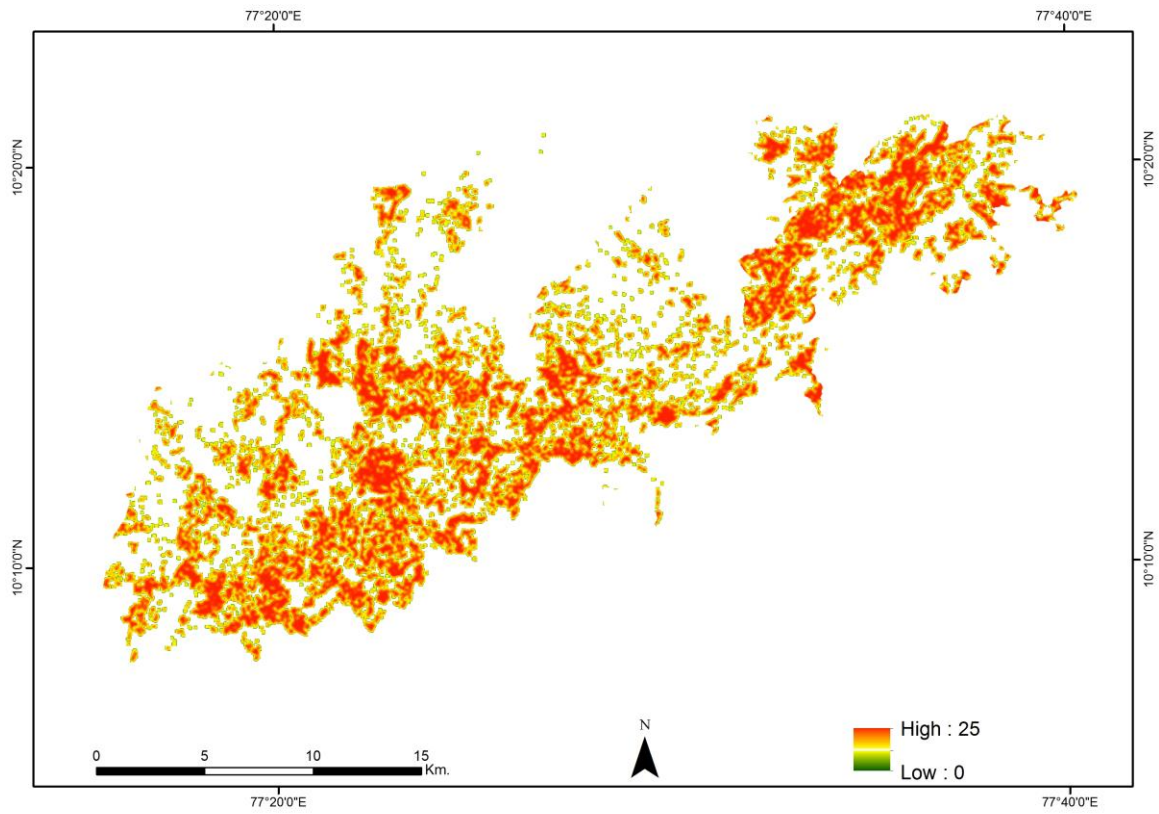

Supplement: S15 Fig — (PDF) [file pone.0190003.s020.pdf]

S16 Fig. Independent variable used for calibration of LRM – 2003 plantation 5 cells window

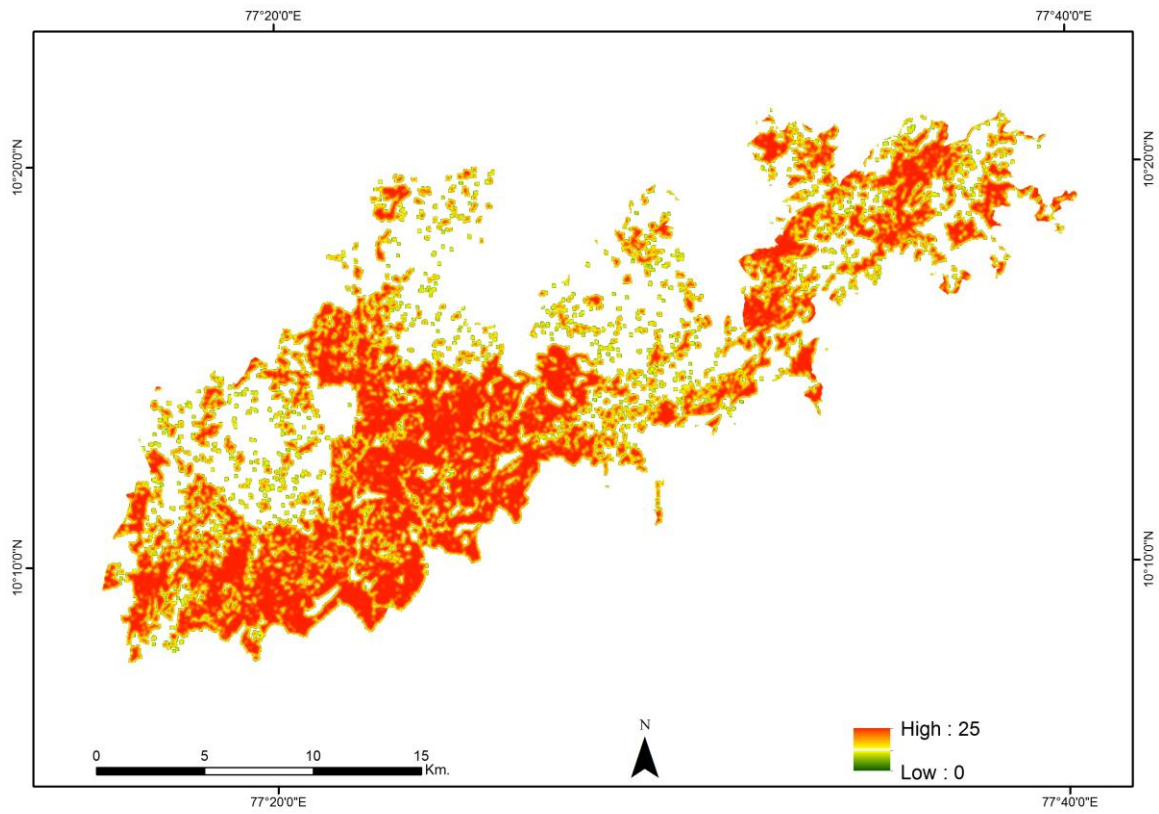

Supplement: S16 Fig — (PDF) [file pone.0190003.s021.pdf]

S17 Fig. Independent variable used for calibration of LRM – 1993 plantation 7 cells window

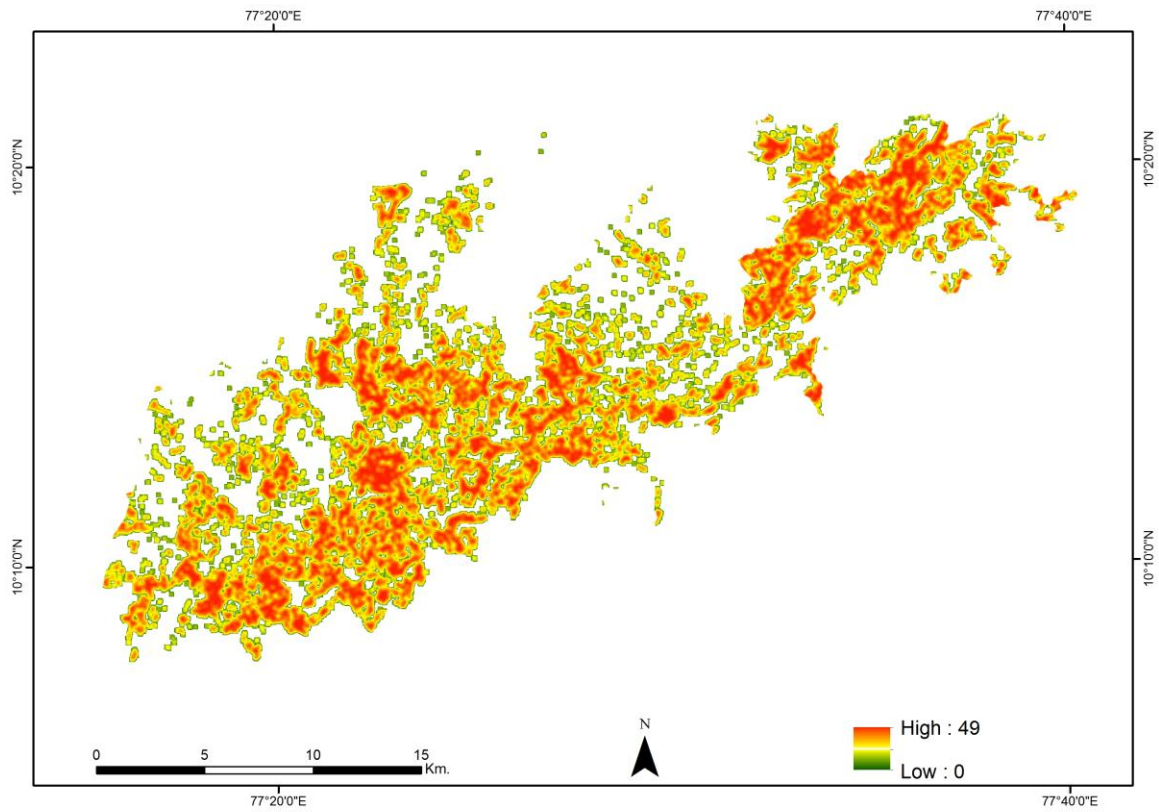

Supplement: S17 Fig — (PDF) [file pone.0190003.s022.pdf]

S18 Fig. Independent variable used for calibration of LRM – 2003 plantation 7 cells window

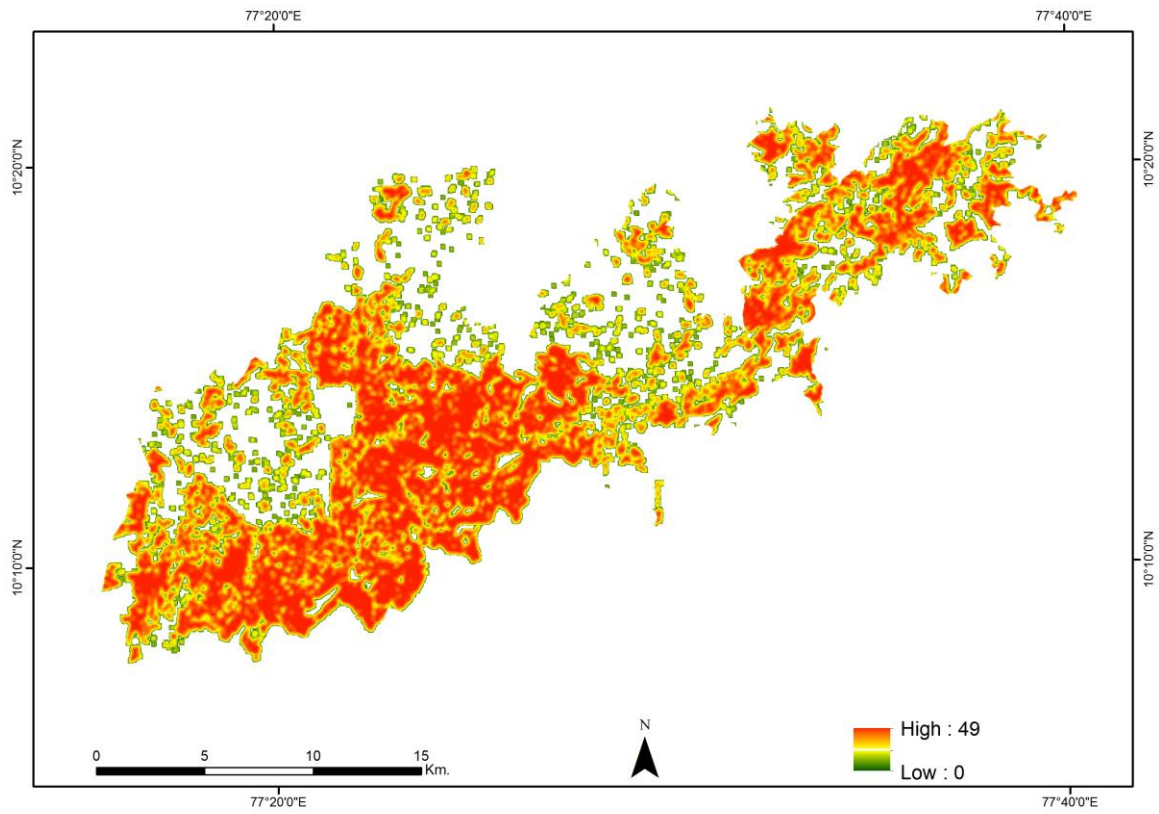

Supplement: S18 Fig — (PDF) [file pone.0190003.s023.pdf]

S19 Fig. Independent variable used for calibration of LRM – 1993 plantation 15 cells window

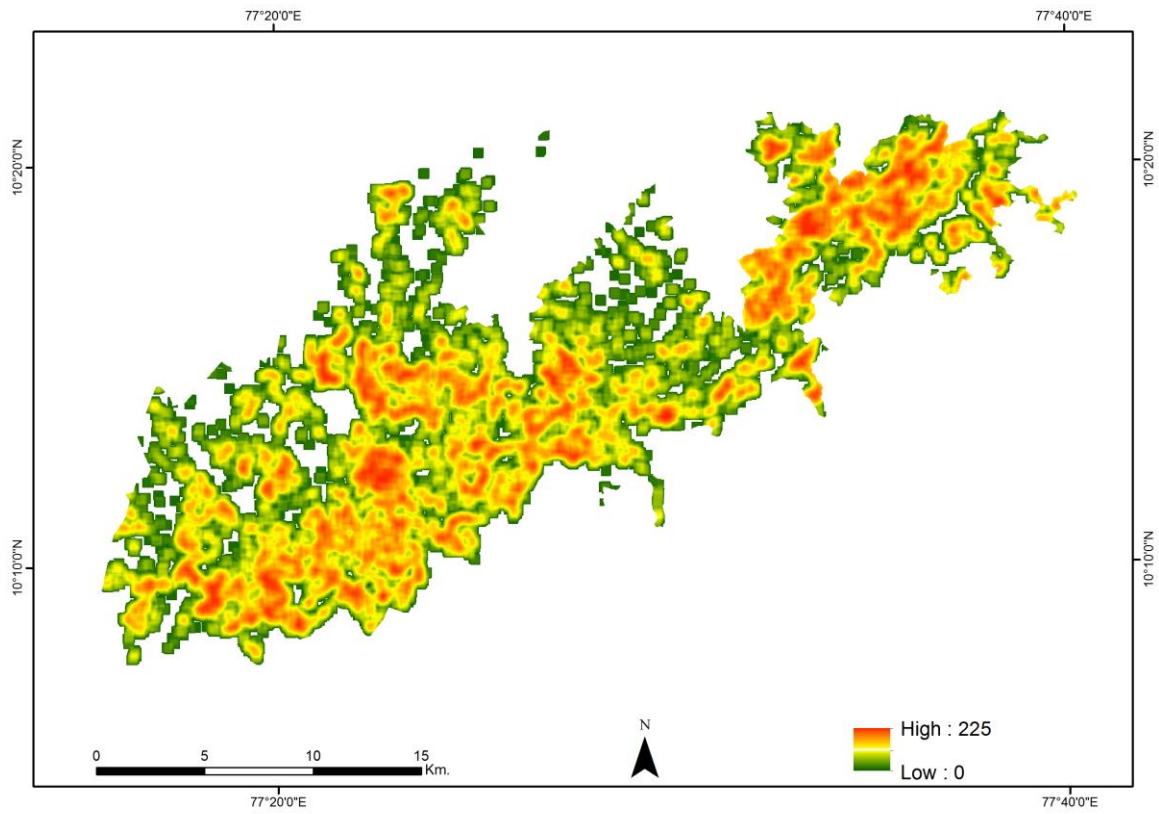

Supplement: S19 Fig — (PDF) [file pone.0190003.s024.pdf]

S20 Fig. Independent variable used for calibration of LRM – 2003 plantation 15 cells window

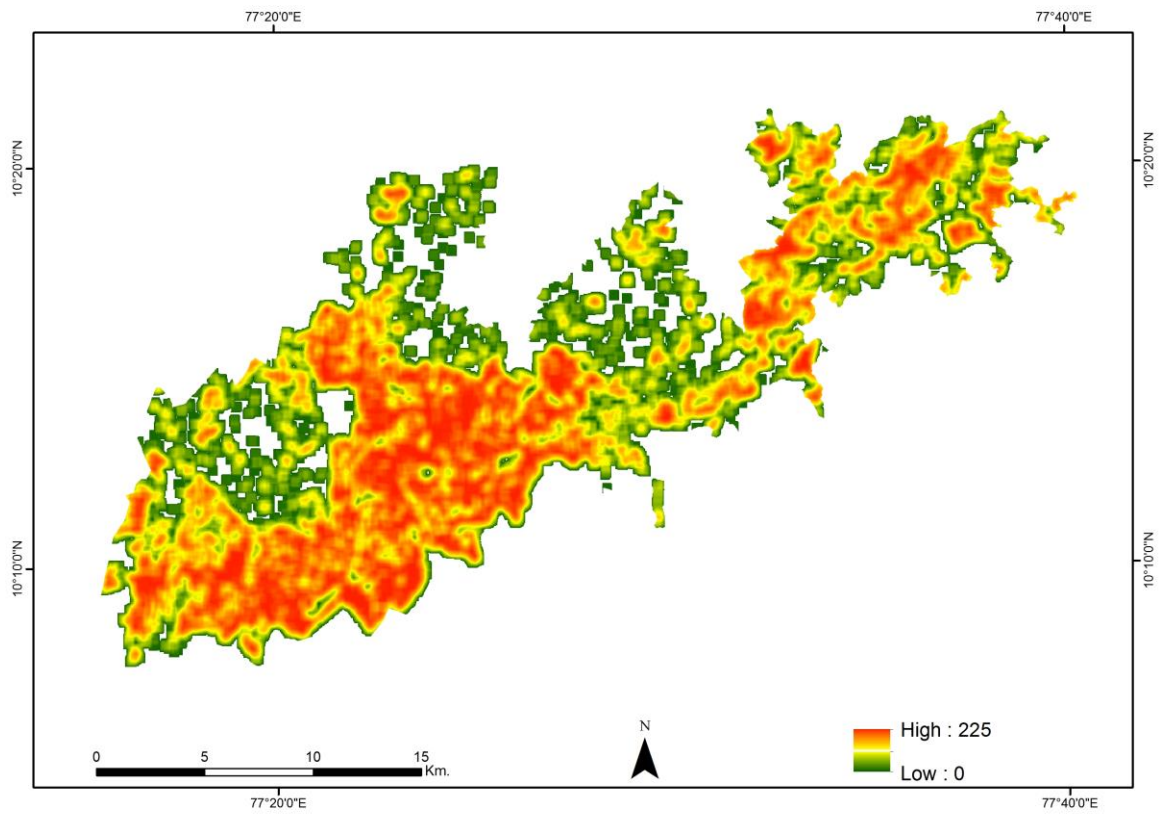

Supplement: S20 Fig — (PDF) [file pone.0190003.s025.pdf]

S21 Fig. Independent variable used for calibration of LRM – 1993 slope variability

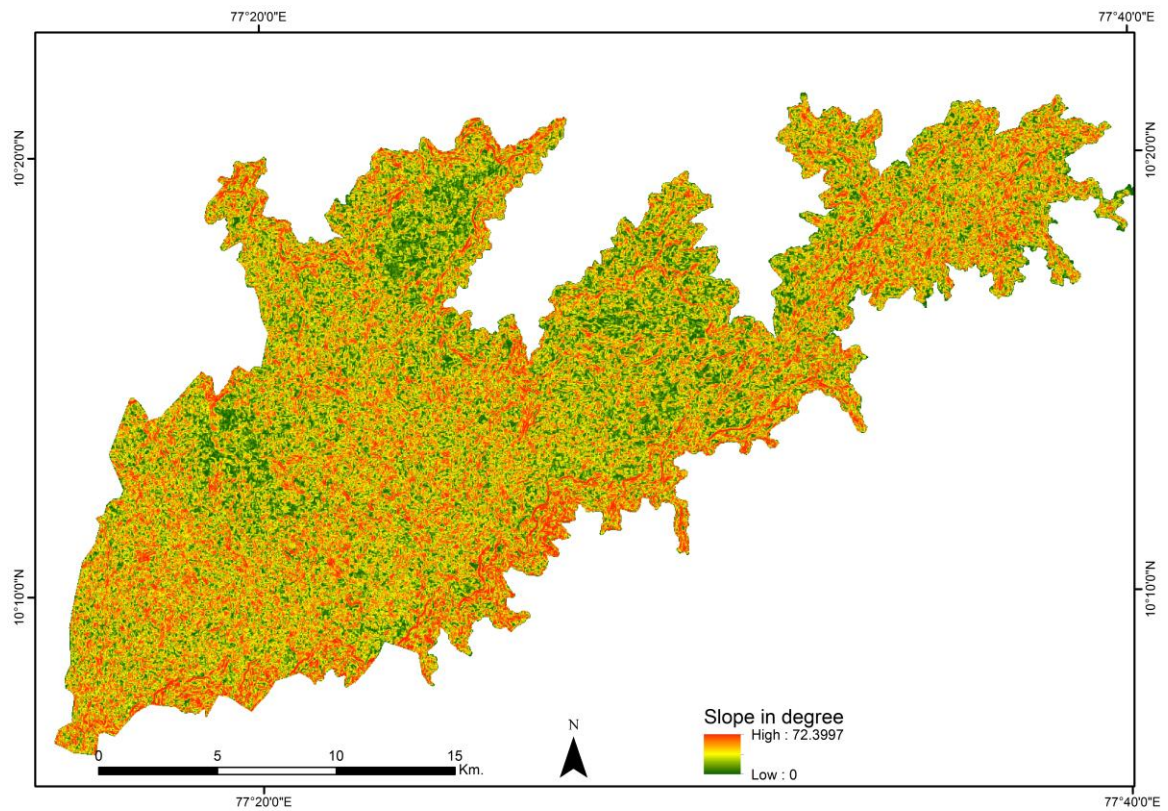

Supplement: S21 Fig — (PDF) [file pone.0190003.s026.pdf]

S22 Fig. Independent variable used for calibration of LRM – 2003 slope variability

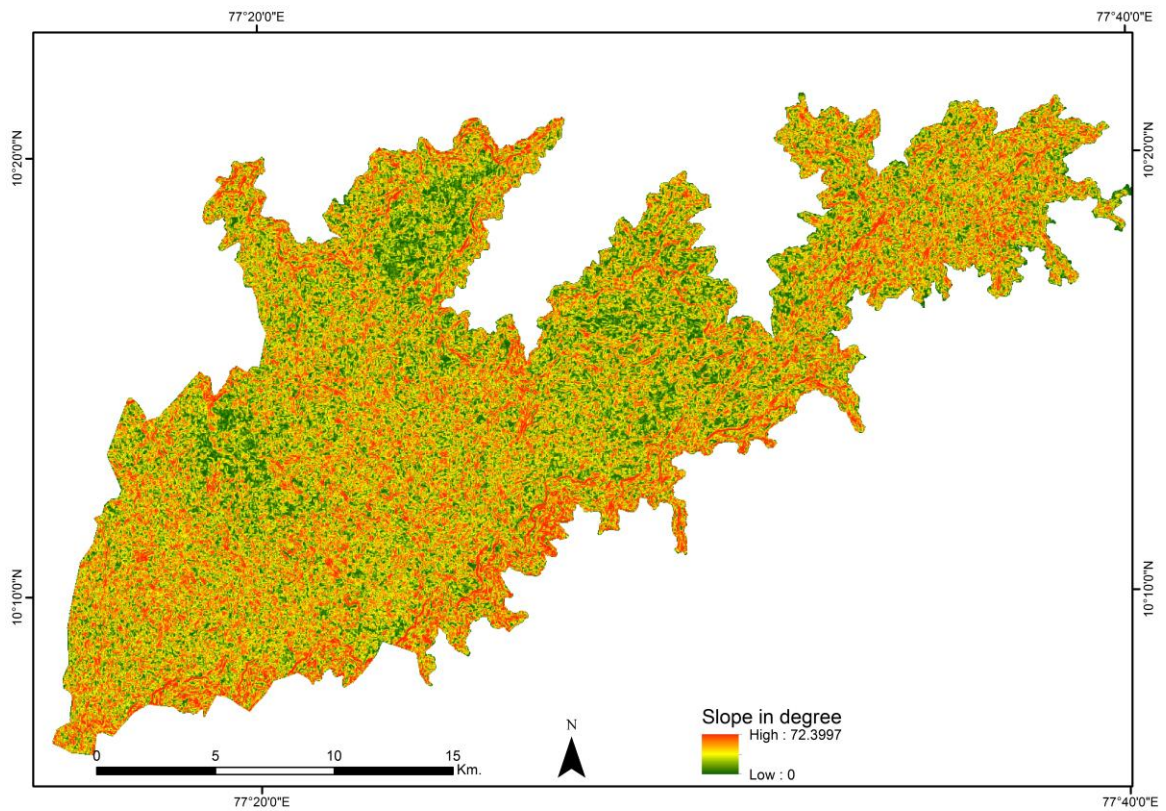

Supplement: S22 Fig — (PDF) [file pone.0190003.s027.pdf]

S24 Fig. Grasslands inside and outside of product area

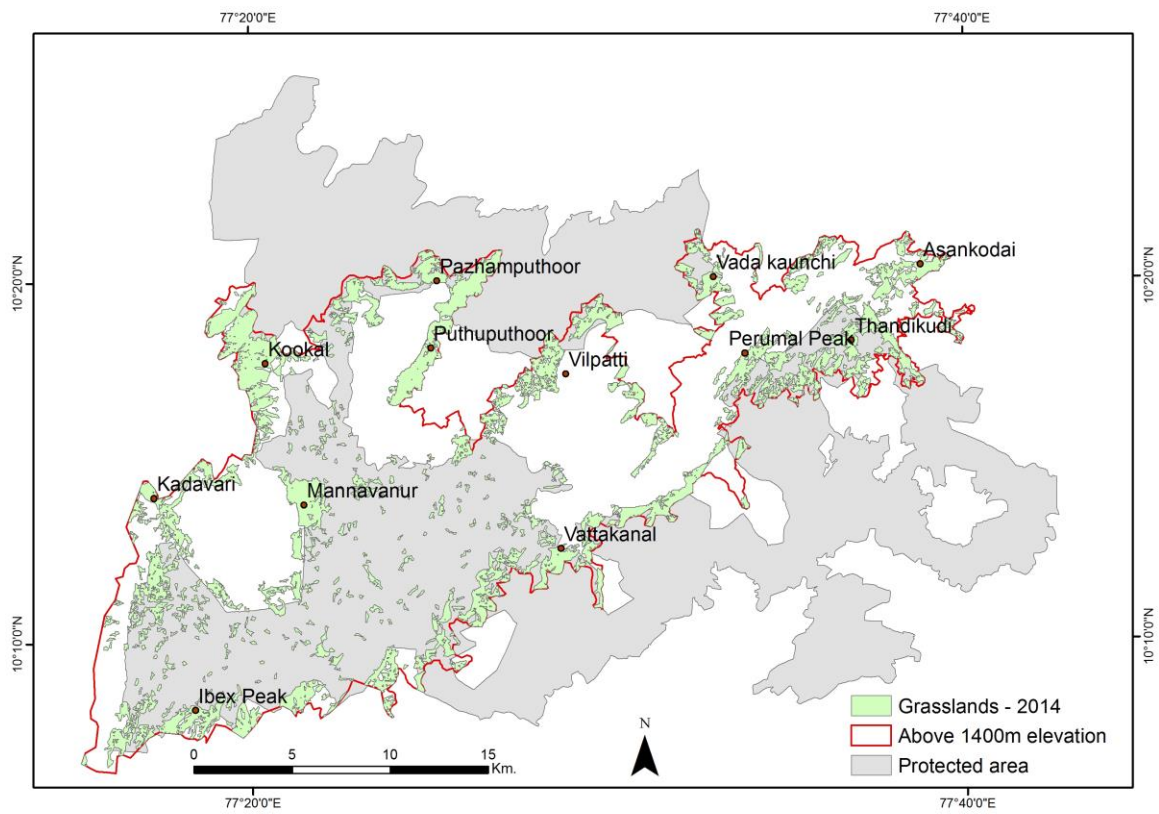

Supplement: S24 Fig — (PDF) [file pone.0190003.s029.pdf]
