# Supplementary material for: Not seeing the grass for the trees: Timber plantations and agriculture shrink tropical montane grassland by two-thirds over four decades in the Palani Hills, a Western Ghats Sky Island
Source: PLoS One. 2018 Jan 10;13(1):e0190003. doi: 10.1371/journal.pone.0190003 (PMC5761842; doi:10.1371/journal.pone.0190003)

S23 Fig: (a) Change in Area-weighted mean patch size (AREA_AM) for grasslands between 1973-2014 (b) Change in AREA_AM for plantations between 1973-2014 (c) Change in AREA_AM for agriculture between 1973-2014.

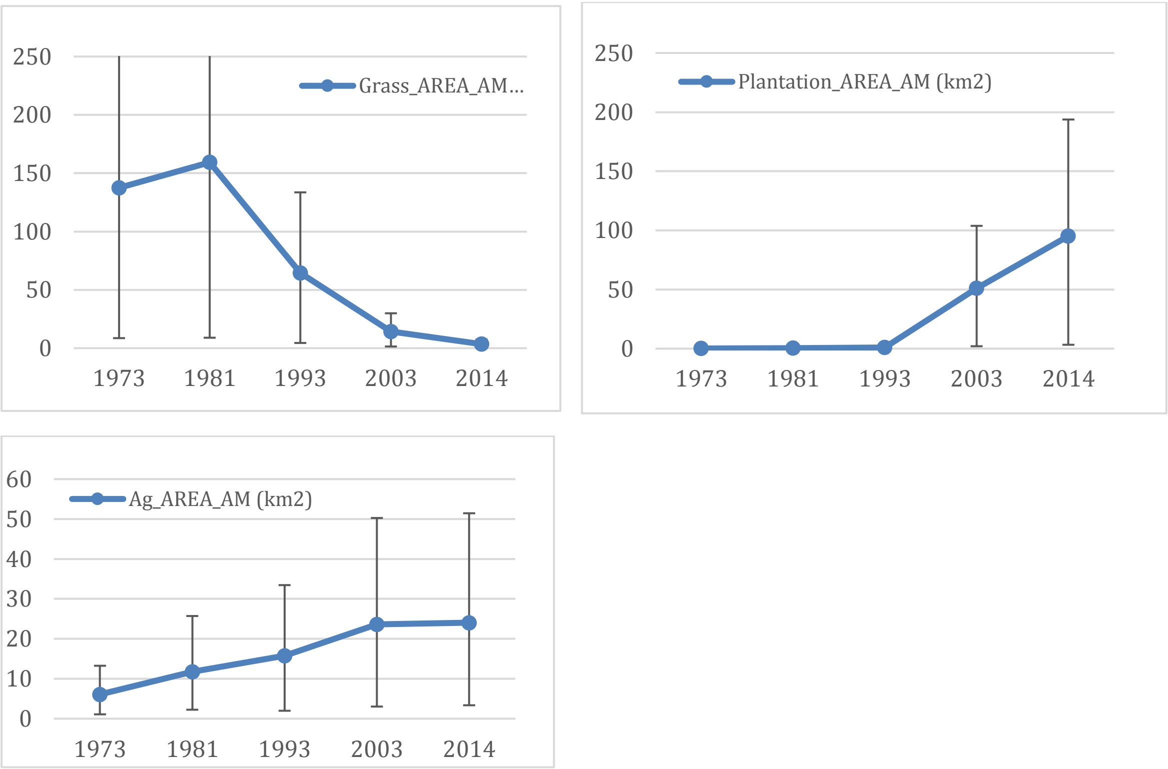

Supplement: S23 Fig — (a) Change in Area-weighted mean patch size (AREA_AM) for grasslands between 1973–2014 (b) Change in AREA_AM for plantations between 1973–2014 (c) Change in AREA_AM for agriculture between 1973–2014. (PDF) [file pone.0190003.s028.pdf]
