# Supplementary material for: Not seeing the grass for the trees: Timber plantations and agriculture shrink tropical montane grassland by two-thirds over four decades in the Palani Hills, a Western Ghats Sky Island
Source: PLoS One. 2018 Jan 10;13(1):e0190003. doi: 10.1371/journal.pone.0190003 (PMC5761842; doi:10.1371/journal.pone.0190003)
Supplement: S1 R Code — (PDF) [file pone.0190003.s030.pdf]

S1 R Code. logistic regression modeling

1993 – 2003 Grassland loss due to agriculture (35 cells moving window of agriculture)

```
setwd('F:/Final/TIFF')
library(raster)
library(rgdal)
library(sp)
library(ROCR)


# LRM inputs for grassland loss to agriculture
grss2agri93to03 <- raster("2003grasslandlossdtagriculture.img")
Agri1993mov35In <- raster("1993agriculture35proportion.img")
Sett1993mov1kmIn <- raster("1993settlements35.rst")
logdist2roadsIn <- raster("Roadlog.tif")
SlopeVar <- raster("Slopevariabilityfloat.tif")
VillageBoundary <- readOGR("F:\\Final\\PalaniHillsVillages.shp")
BeatBoundary <- readOGR("F:\\Final\\KodaikanalBeatDissolve.shp")

crs(SlopeVar)
crs(logdist2roadsIn)
crs(Sett1993mov1kmIn)
crs(Agri1993mov35In)
crs(grss2agri93to03)

Agri1993mov35In <- projectRaster(Agri1993mov35In, crs=crs(SlopeVar))
Sett1993mov1kmIn <- projectRaster(Sett1993mov1kmIn, crs=crs(SlopeVar))
grss2agri93to03 <- projectRaster(grss2agri93to03, crs=crs(SlopeVar), method="ngb")
```

```
# Convert grassland change raster to point layer
grss2agri93to03Spdf <- rasterToPoints(grss2agri93to03, spatial=T)

# Change column name and remove zeroes from SpDF
names(grss2agri93to03Spdf) <- c("losstoagri93_03")
grss2agri93to03Spdf_12 <- grss2agri93to03Spdf [grss2agri93to03Spdf@data$losstoagri93_03 != 0, ]

# Extract values from predictor rasters
grss2agri93to03Spdf_12$Agricmov35 <- extract(Agri1993mov35In, grss2agri93to03Spdf_12)
grss2agri93to03Spdf_12$logd2road <- extract(logdist2roadsIn, grss2agri93to03Spdf_12)
grss2agri93to03Spdf_12$settwin1k <- extract(Sett1993mov1kmIn, grss2agri93to03Spdf_12)
grss2agri93to03Spdf_12$slopevarb  <- extract(SlopeVar, grss2agri93to03Spdf_12)
# grss2agri93to03Spdf_12$villagecd <- as.list(over(grss2agri93to03Spdf_12, VillageBoundary))

# Recode grassland no-change from 2 to 0
grss2agri93to03Spdf_12$losstoagri93_03RC <- ifelse(grss2agri93to03Spdf_12@data$losstoagri93_03 == 2, 0, grss2agri93to03Spdf_12@data$losstoagri93_03)

# adding xy information, remove NAs from data, remove old column
grss2agri93to03Spdf_12$lat <- grss2agri93to03Spdf_12@coords[,2]
grss2agri93to03Spdf_12$lon <- grss2agri93to03Spdf_12@coords[,1]

grss93to03Spdf_10 <- na.omit(grss2agri93to03Spdf_12@data)
grss93to03Spdf_10 <- grss93to03Spdf_10[,c(2:8)]
```

```
# grss93to03Spdf_10VC <- na.omit(grss2agri93to03Spdf_12@data)
# grss93to03Spdf_10VC <- grss93to03Spdf_10VC[,c(2:5, 7:9)]


# Checking for correlations
head(grss93to03Spdf_10)
cor(grss93to03Spdf_10)

#sample

nrow(grss93to03Spdf_10)

(278954 /100)*80

(278954 /100)*20


#Eighty percentage sample for train


train.9303agri <- grss93to03Spdf_10[sample(1:nrow(grss93to03Spdf_10), 223163,replace=FALSE),]
nrow(train.9303agri)

#Eighty percentage sample for test

test.9303agri<-grss93to03Spdf_10[setdiff(rownames(grss93to03Spdf_10), rownames(train.9303agri)), ]
nrow(test.9303agri)
head(test.9303agri)
```

```
#LRM
grss2agri93to03.binom <- glm(losstoagri93_03RC ~ settwin1k + logd2road + Agricmov35
        + slopevarb, family=binomial(link = 'logit'), data=train.9303agri)
summary(grss2agri93to03.binom)


# Create empty dataframe to store predictions and actuals
library(AUC)
test.9303.rocIn <- data.frame(matrix(vector(), nrow(test.9303agri), 2,
        dimnames=list(c(), c("True.Fate", "Predictions"))), stringsAsFactors=F)
test.9303.rocIn$True.Fate <- as.factor(test.9303agri$losstoagri93_03RC)
test.9303.rocIn$Predictions <- predict.glm(grss2agri93to03.binom, newdata=test.9303agri,
        type='response')

test.9303.roc <- roc(test.9303.rocIn$Predictions, test.9303.rocIn$True.Fate)
test.9303.auc <- auc(test.9303.roc)


save.image("F:\\LRMfinalResults\\1993_2003GrasslandLossDTagriculture_35cells.RData")
```

1993 – 2003 Grassland loss due to agriculture (7 cells moving window of agriculture)

```
setwd('F:/Final/TIFF')
library(raster)
library(rgdal)
library(sp)
library(ROCR)
```

```
# LRM inputs for grassland loss to agriculture
grss2agri93to03 <- raster("2003grasslandlossdtagriculture.img")
Agri1993mov7In <- raster("1993agriculture7.tif")
Sett1993mov1kmIn <- raster("1993settlements35.rst")
logdist2roadsIn <- raster("Roadlog.tif")
SlopeVar <- raster("Slopevariabilityfloat.tif")
VillageBoundary <- readOGR("F:\\Final\\PalaniHillsVillages.shp")
BeatBoundary <- readOGR("F:\\Final\\KodaikanalBeatDissolve.shp")

crs(SlopeVar)
crs(logdist2roadsIn)
crs(Sett1993mov1kmIn)
crs(Agri1993mov7In)
crs(grss2agri93to03)



Sett1993mov1kmIn <- projectRaster(Sett1993mov1kmIn, crs=crs(SlopeVar))
grss2agri93to03 <- projectRaster(grss2agri93to03, crs=crs(SlopeVar), method="ngb")



# Convert grassland change raster to point layer
grss2agri93to03Spdf <- rasterToPoints(grss2agri93to03, spatial=T)

# Change column name and remove zeroes from SpDF
names(grss2agri93to03Spdf) <- c("losstoagri93_03")
grss2agri93to03Spdf_12 <- grss2agri93to03Spdf [grss2agri93to03Spdf@data$losstoagri93_03 != 0, ]
```

```
# Extract values from predictor rasters
grss2agri93to03Spdf_12$Agricmov7 <- extract(Agri1993mov7In, grss2agri93to03Spdf_12)
grss2agri93to03Spdf_12$logd2road <- extract(logdist2roadsIn, grss2agri93to03Spdf_12)
grss2agri93to03Spdf_12$settwin1k <- extract(Sett1993mov1kmIn, grss2agri93to03Spdf_12)
grss2agri93to03Spdf_12$slopevarb <- extract(SlopeVar, grss2agri93to03Spdf_12)
# grss2agri93to03Spdf_12$villagecd <- as.list(over(grss2agri93to03Spdf_12, VillageBoundary))


# Recode grassland no-change from 2 to 0
grss2agri93to03Spdf_12$losstoagri93_03RC <- ifelse(grss2agri93to03Spdf_12@data$losstoagri93_03 == 2, 0, grss2agri93to03Spdf_12@data$losstoagri93_03)


# adding xy information, remove NAs from data, remove old column
grss2agri93to03Spdf_12$lat <- grss2agri93to03Spdf_12@coords[,2]
grss2agri93to03Spdf_12$lon <- grss2agri93to03Spdf_12@coords[,1]

grss93to03Spdf_10 <- na.omit(grss2agri93to03Spdf_12@data)
grss93to03Spdf_10 <- grss93to03Spdf_10[,c(2:8)]

# grss93to03Spdf_10VC <- na.omit(grss2agri93to03Spdf_12@data)
# grss93to03Spdf_10VC <- grss93to03Spdf_10VC[,c(2:5, 7:9)]


# Checking for correlations
head(grss93to03Spdf_10)
cor(grss93to03Spdf_10)
```

```
#sample

nrow(grss93to03Spdf_10)

(278954 /100)*80

(278954 /100)*20


#Eighty percentage sample for train


train.9303agri <- grss93to03Spdf_10[sample(1:nrow(grss93to03Spdf_10),
223163,replace=FALSE),]
nrow(train.9303agri)

#Eighty percentage sample for test

test.9303agri<-grss93to03Spdf_10[setdiff(rownames(grss93to03Spdf_10),
rownames(train.9303agri)), ]
nrow(test.9303agri)
head(test.9303agri)

#LRM
grss2agri93to03.binom <- glm(losstoagri93_03RC ~ settwin1k + logd2road +
Agricmov7
                 + slopevarb, family=binomial(link = 'logit'), data=train.9303agri)
summary(grss2agri93to03.binom)
```

```
# Create empty dataframe to store predictions and actuals
library(AUC)
test.9303.rocIn <- data.frame(matrix(vector(), nrow(test.9303agri), 2,
                        dimnames=list(c(),   c("True.Fate",   "Predictions"))),
stringsAsFactors=F)
test.9303.rocIn$True.Fate <- as.factor(test.9303agri$losstoagri93_03RC)
test.9303.rocIn$Predictions      <-      predict.glm(grss2agri93to03.binom,
newdata=test.9303agri,
                          type='response')

test.9303.roc <- roc(test.9303.rocIn$Predictions, test.9303.rocIn$True.Fate)
test.9303.auc <- auc(test.9303.roc)


save.image(file='F:\\LRMfinalResults\\1993_2003GrasslandLossDTagriculture.RData')
```

1993 – 2003 Grassland loss due to plantation (5 cells moving window of plantation)

```
setwd('F:/Final/TIFF')
library(raster)
library(rgdal)
library(sp)
library(ROCR)


# LRM inputs for grassland loss to plantculture
grss2plant93to03 <- raster("2003grasslandlossdtplantations.img")
Plant1993mov5In <- raster("1993plantation5.tif")
Sett1993mov1kmIn <- raster("1993settlements35.rst")
logdist2roadsIn <- raster("Roadlog.tif")
SlopeVar <- raster("Slopevariabilityfloat.tif")
```

```
VillageBoundary <- readOGR("F:\\Final\\PalaniHillsVillages.shp")
BeatBoundary <- readOGR("F:\\Final\\KodaikanalBeatDissolve.shp")

crs(SlopeVar)
crs(logdist2roadsIn)
crs(Sett1993mov1kmIn)
crs(Plant1993mov5In)
crs(grss2plant93to03)

Plant1993mov5In <- projectRaster(Plant1993mov5In, crs=crs(SlopeVar))
Sett1993mov1kmIn <- projectRaster(Sett1993mov1kmIn, crs=crs(SlopeVar))
grss2plant93to03 <- projectRaster(grss2plant93to03, crs=crs(SlopeVar), method="ngb")

# crs(grss2plant93to03) <- crs(logdist2roadsIn)

# Convert grassland change raster to point layer
grss2plant93to03Spdf <- rasterToPoints(grss2plant93to03, spatial=T)

# Change column name and remove zeroes from SpDF
names(grss2plant93to03Spdf) <- c("losstoplant93_03")
grss2plant93to03Spdf_12 <- grss2plant93to03Spdf [grss2plant93to03Spdf@data$losstoplant93_03 != 0, ]

# Extract values from predictor rasters
grss2plant93to03Spdf_12$Plant1993mov5In <- extract(Plant1993mov5In, grss2plant93to03Spdf_12)
grss2plant93to03Spdf_12$logd2road <- extract(logdist2roadsIn, grss2plant93to03Spdf_12)
grss2plant93to03Spdf_12$settwin1k <- extract(Sett1993mov1kmIn, grss2plant93to03Spdf_12)
grss2plant93to03Spdf_12$slopevarb <- extract(SlopeVar, grss2plant93to03Spdf_12)
```

```
# grss2plant93to03Spdf_12$villagecd <- as.list(over(grss2plant93to03Spdf_12, VillageBoundary))

# Recode grassland no-change from 2 to 0
grss2plant93to03Spdf_12$losstoplant93_03RC <- ifelse(grss2plant93to03Spdf_12@data$losstoplant93_03 == 2, 0, grss2plant93to03Spdf_12@data$losstoplant93_03)

# adding xy information, remove NAs from data, remove old column
grss2plant93to03Spdf_12$lat <- grss2plant93to03Spdf_12@coords[,2]
grss2plant93to03Spdf_12$lon <- grss2plant93to03Spdf_12@coords[,1]

grss93to03Spdf_10 <- na.omit(grss2plant93to03Spdf_12@data)
grss93to03Spdf_10 <- grss93to03Spdf_10[,c(2:8)]

# grss93to03Spdf_10VC <- na.omit(grss2plant93to03Spdf_12@data)
# grss93to03Spdf_10VC <- grss93to03Spdf_10VC[,c(2:5, 7:9)]

# Checking for correlations
head(grss93to03Spdf_10)
cor(grss93to03Spdf_10)

#sample

nrow(grss93to03Spdf_10)

(305717 /100)*80
```

```
(305717 /100)*20


# Eighty percentage sample for train

train.9303plant <- grss93to03Spdf_10[sample(1:nrow(grss93to03Spdf_10), 244574,replace=FALSE),]
head(train.9303plant)
nrow(train.9303plant)

# Twenty percentage sample for test
test.9303plant <- grss93to03Spdf_10[setdiff(rownames(grss93to03Spdf_10), rownames(train.9303plant)), ]
nrow(test.9303plant)
head(test.9303plant)

# LRM
grss2plant93to03.binom <- glm(losstoplant93_03RC ~ settwin1k + logd2road + Plant1993mov5In+ slopevarb, family=binomial(link='logit'), data=train.9303plant)
summary(grss2plant93to03.binom)



# Create empty dataframe to store predictions and actuals
library(AUC)
test.9303.rocIn <- data.frame(matrix(vector(), nrow(test.9303plant), 2,
                              dimnames=list(c(), c("True.Fate", "Predictions"))), stringsAsFactors=F)
test.9303.rocIn$True.Fate <- as.factor(test.9303plant$losstoplant93_03RC)
test.9303.rocIn$Predictions <- predict.glm(grss2plant93to03.binom, newdata=test.9303plant,
                                   type='response')
```

```
test.9303.roc <- roc(test.9303.rocIn$Predictions, test.9303.rocIn$True.Fate)
test.9303.auc <- auc(test.9303.roc)


save.image(file='F:\\LRMfinalResults\\1993_2003GrasslandLossDTplantations.RData'
)
```

1993 – 2003 Grassland loss due to plantation (15 cells moving window of plantation)

```
setwd('F:/Final/TIFF')
library(raster)
library(rgdal)
library(sp)
library(ROCR)


# LRM inputs for grassland loss to plantculture
grss2plant93to03 <- raster("2003grasslandlossdtplantations.img")
Plant1993mov15In <- raster("1993plantation15.tif")
Sett1993mov1kmIn <- raster("1993settlements35.rst")
logdist2roadsIn <- raster("Roadlog.tif")
SlopeVar <- raster("Slopevariabilityfloat.tif")
VillageBoundary <- readOGR("F:\\Final\\PalaniHillsVillages.shp")
BeatBoundary <- readOGR("F:\\Final\\KodaikanalBeatDissolve.shp")

crs(SlopeVar)
crs(logdist2roadsIn)
crs(Sett1993mov1kmIn)
crs(Plant1993mov15In)
crs(grss2plant93to03)
```

```
Plant1993mov15In <- projectRaster(Plant1993mov15In, crs=crs(SlopeVar))

Sett1993mov1kmIn <- projectRaster(Sett1993mov1kmIn, crs=crs(SlopeVar))

grss2plant93to03 <- projectRaster(grss2plant93to03, crs=crs(SlopeVar), method="ngb")


# crs(grss2plant93to03) <- crs(logdist2roadsIn)


# Convert grassland change raster to point layer

grss2plant93to03Spdf <- rasterToPoints(grss2plant93to03, spatial=T)


# Change column name and remove zeroes from SpDF

names(grss2plant93to03Spdf) <- c("losstoplant93_03")

grss2plant93to03Spdf_12 <- grss2plant93to03Spdf [grss2plant93to03Spdf@data$losstoplant93_03 != 0, ]


# Extract values from predictor rasters

grss2plant93to03Spdf_12$Plant1993mov15In <- extract(Plant1993mov15In, grss2plant93to03Spdf_12)

grss2plant93to03Spdf_12$logd2road <- extract(logdist2roadsIn, grss2plant93to03Spdf_12)

grss2plant93to03Spdf_12$settwin1k <- extract(Sett1993mov1kmIn, grss2plant93to03Spdf_12)

grss2plant93to03Spdf_12$slopevarb <- extract(SlopeVar, grss2plant93to03Spdf_12)

# grss2plant93to03Spdf_12$villagecd <- as.list(over(grss2plant93to03Spdf_12, VillageBoundary))



# Recode grassland no-change from 2 to 0

grss2plant93to03Spdf_12$losstoplant93_03RC <- ifelse(grss2plant93to03Spdf_12@data$losstoplant93_03 == 2, 0, grss2plant93to03Spdf_12@data$losstoplant93_03)
```

```
# adding xy information, remove NAs from data, remove old column
grss2plant93to03Spdf_12$lat <- grss2plant93to03Spdf_12@coords[,2]
grss2plant93to03Spdf_12$lon <- grss2plant93to03Spdf_12@coords[,1]

grss93to03Spdf_10 <- na.omit(grss2plant93to03Spdf_12@data)
grss93to03Spdf_10 <- grss93to03Spdf_10[,c(2:8)]

# grss93to03Spdf_10VC <- na.omit(grss2plant93to03Spdf_12@data)
# grss93to03Spdf_10VC <- grss93to03Spdf_10VC[,c(2:5, 7:9)]


# Checking for correlations
head(grss93to03Spdf_10)
cor(grss93to03Spdf_10)

#sample

nrow(grss93to03Spdf_10)

(305717 /100)*80

(305717 /100)*20


# Eighty percentage sample for train

train.9303plant <- grss93to03Spdf_10[sample(1:nrow(grss93to03Spdf_10),
244574,replace=FALSE),]
head(train.9303plant)
nrow(train.9303plant)
```

```
# Twenty percentage sample for test
test.9303plant <- grss93to03Spdf_10[setdiff(rownames(grss93to03Spdf_10), rownames(train.9303plant)), ]
nrow(test.9303plant)
head(test.9303plant)

# LRM
grss2plant93to03.binom <- glm(losstoplant93_03RC ~ settwin1k + logd2road + Plant1993mov15In+ slopevarb, family=binomial(link='logit'), data=train.9303plant)
summary(grss2plant93to03.binom)

# Create empty dataframe to store predictions and actuals
library(AUC)
test.9303.rocIn <- data.frame(matrix(vector(), nrow(test.9303plant), 2,
                        dimnames=list(c(), c("True.Fate", "Predictions"))), stringsAsFactors=F)
test.9303.rocIn$True.Fate <- as.factor(test.9303plant$losstoplant93_03RC)
test.9303.rocIn$Predictions <- predict.glm(grss2plant93to03.binom, newdata=test.9303plant,
                           type='response')

test.9303.roc <- roc(test.9303.rocIn$Predictions, test.9303.rocIn$True.Fate)
test.9303.auc <- auc(test.9303.roc)

save.image(file='F:\\LRMfinalResults\\1993_2003GrasslandLossDTplantations_15cells.RData')
```

2003 – 2014 Grassland loss due to agriculture (35 cells moving window of agriculture)

```
setwd('F:/Final/TIFF')
library(raster)
library(rgdal)
library(sp)
library(ROCR)


# LRM inputs for grassland loss to agriculture
grss2agri03to14 <- raster("2014grasslandlossdtagriculture.img")
Agri2003mov7In <- raster("2003Agriculture.rst")
Sett2003mov1kmIn <- raster("2003settlemets35.tif")
logdist2roadsIn <- raster("Roadlog.tif")
SlopeVar <- raster("Slopevariabilityfloat.tif")
VillageBoundary <- readOGR("F:\\Final\\PalaniHillsVillages.shp")
BeatBoundary <- readOGR("F:\\Final\\KodaikanalBeatDissolve.shp")

crs(SlopeVar)
crs(logdist2roadsIn)
crs(Sett2003mov1kmIn)
crs(Agri2003mov7In)
crs(grss2agri03to14)


Agri2003mov7In <- projectRaster(Agri2003mov7In, crs=crs(SlopeVar))
grss2agri03to14 <- projectRaster(grss2agri03to14, crs=crs(SlopeVar), method="ngb")


# Convert grassland change raster to point layer
grss2agri03to14Spdf <- rasterToPoints(grss2agri03to14, spatial=T)
```

```
# Change column name and remove zeroes from SpDF
names(grss2agri03to14Spdf) <- c("losstoagri03_14")
grss2agri03to14Spdf_12 <- grss2agri03to14Spdf [grss2agri03to14Spdf@data$losstoagri03_14 != 0, ]

# Extract values from predictor rasters
grss2agri03to14Spdf_12$Agricmov7 <- extract(Agri2003mov7In, grss2agri03to14Spdf_12)
grss2agri03to14Spdf_12$logd2road <- extract(logdist2roadsIn, grss2agri03to14Spdf_12)
grss2agri03to14Spdf_12$settwin1k <- extract(Sett2003mov1kmIn, grss2agri03to14Spdf_12)
grss2agri03to14Spdf_12$slopevarb <- extract(SlopeVar, grss2agri03to14Spdf_12)
# grss2agri03to14Spdf_12$villagecd <- as.list(over(grss2agri03to14Spdf_12, VillageBoundary))


# Recode grassland no-change from 2 to 0
grss2agri03to14Spdf_12$losstoagri03_14RC <- ifelse(grss2agri03to14Spdf_12@data$losstoagri03_14 == 2, 0, grss2agri03to14Spdf_12@data$losstoagri03_14)


# adding xy information, remove NAs from data, remove old column
grss2agri03to14Spdf_12$lat <- grss2agri03to14Spdf_12@coords[,2]
grss2agri03to14Spdf_12$lon <- grss2agri03to14Spdf_12@coords[,1]

grss03to14Spdf_10 <- na.omit(grss2agri03to14Spdf_12@data)
grss03to14Spdf_10 <- grss03to14Spdf_10[,c(2:8)]

# grss03to14Spdf_10VC <- na.omit(grss2agri03to14Spdf_12@data)
# grss03to14Spdf_10VC <- grss03to14Spdf_10VC[,c(2:5, 7:9)]
```

```
# Checking for correlations
head(grss03to14Spdf_10)
cor(grss03to14Spdf_10)

#sample

nrow(grss03to14Spdf_10)

(191525 /100)*80

(191525 /100)*20


#Eighty percentage sample for train


train.0314agri <- grss03to14Spdf_10[sample(1:nrow(grss03to14Spdf_10), 153220,replace=FALSE),]
nrow(train.0314agri)

#Eighty percentage sample for test

test.0314agri<-grss03to14Spdf_10[setdiff(rownames(grss03to14Spdf_10), rownames(train.0314agri)), ]
nrow(test.0314agri)
head(test.0314agri)

#LRM
grss2agri03to14.binom <- glm(losstoagri03_14RC ~ settwin1k + logd2road + Agricmov7
```

```
                + slopevarb, family=binomial(link = 'logit'), data=train.0314agri)
summary(grss2agri03to14.binom)


# Create empty dataframe to store predictions and actuals
library(AUC)
test.0314.rocIn <- data.frame(matrix(vector(), nrow(test.0314agri), 2,
                    dimnames=list(c(),     c("True.Fate",     "Predictions"))),
stringsAsFactors=F)
test.0314.rocIn$True.Fate <- as.factor(test.0314agri$losstoagri03_14RC)
test.0314.rocIn$Predictions          <-          predict.glm(grss2agri03to14.binom,
newdata=test.0314agri,
                      type='response')

test.0314.roc <- roc(test.0314.rocIn$Predictions, test.0314.rocIn$True.Fate)
test.0314.auc <- auc(test.0314.roc)


save.image(file='F:\\LRMfinalResults\\2003_2014GrasslandLossDTagriculture.RData')
```

2003 – 2014 Grassland loss due to plantation (5 cells moving window of plantation)

```
setwd('F:/Final/TIFF')
library(raster)
library(rgdal)
library(sp)
library(ROCR)
```

```
# LRM inputs for grassland loss to plantculture
grss2plant03to14 <- raster("2014grasslandlossdtplantations.img")
Plant2003mov5In <- raster("Plantationmovingwindow5.tif")
Sett2003mov1kmIn <- raster("2003settlemets35.tif")
logdist2roadsIn <- raster("Roadlog.tif")
SlopeVar <- raster("Slopevariabilityfloat.tif")
VillageBoundary <- readOGR("F:\\Final\\PalaniHillsVillages.shp")
BeatBoundary <- readOGR("F:\\Final\\KodaikanalBeatDissolve.shp")

crs(SlopeVar)
crs(logdist2roadsIn)
crs(Sett2003mov1kmIn)
crs(Plant2003mov5In)
crs(grss2plant03to14)

Plant2003mov5In <- projectRaster(Plant2003mov5In, crs=crs(SlopeVar))
Sett2003mov1kmIn <- projectRaster(Sett2003mov1kmIn, crs=crs(SlopeVar))
grss2plant03to14 <- projectRaster(grss2plant03to14, crs=crs(SlopeVar),
method="ngb")

# crs(grss2plant03to14) <- crs(logdist2roadsIn)

# Convert grassland change raster to point layer
grss2plant03to14Spdf <- rasterToPoints(grss2plant03to14, spatial=T)

# Change column name and remove zeroes from SpDF
names(grss2plant03to14Spdf) <- c("losstoplant03_14")
grss2plant03to14Spdf_12 <- grss2plant03to14Spdf
[grss2plant03to14Spdf@data$losstoplant03_14 != 0, ]

# Extract values from predictor rasters
```

```
grss2plant03to14Spdf_12$Plant2003mov5In <- extract(Plant2003mov5In, grss2plant03to14Spdf_12)
grss2plant03to14Spdf_12$logd2road <- extract(logdist2roadsIn, grss2plant03to14Spdf_12)
grss2plant03to14Spdf_12$settwin1k <- extract(Sett2003mov1kmIn, grss2plant03to14Spdf_12)
grss2plant03to14Spdf_12$slopevarb <- extract(SlopeVar, grss2plant03to14Spdf_12)
# grss2plant03to14Spdf_12$villagecd <- as.list(over(grss2plant03to14Spdf_12, VillageBoundary))


# Recode grassland no-change from 2 to 0
grss2plant03to14Spdf_12$losstoplant03_14RC <- ifelse(grss2plant03to14Spdf_12@data$losstoplant03_14 == 2, 0, grss2plant03to14Spdf_12@data$losstoplant03_14)


# adding xy information, remove NAs from data, remove old column
grss2plant03to14Spdf_12$lat <- grss2plant03to14Spdf_12@coords[,2]
grss2plant03to14Spdf_12$lon <- grss2plant03to14Spdf_12@coords[,1]

 grss03to14Spdf_10 <- na.omit(grss2plant03to14Spdf_12@data)
 grss03to14Spdf_10 <- grss03to14Spdf_10[,c(2:8)]

# grss03to14Spdf_10VC <- na.omit(grss2plant03to14Spdf_12@data)
# grss03to14Spdf_10VC <- grss03to14Spdf_10VC[,c(2:5, 7:9)]


# Checking for correlations
head( grss03to14Spdf_10)
cor( grss03to14Spdf_10)
```

```
#sample

nrow( grss03to14Spdf_10)

(234207 /100)*80

(234207 /100)*20


# Eighty percentage sample for train

train.0314plant <- grss03to14Spdf_10[sample(1:nrow( grss03to14Spdf_10), 187366,replace=FALSE),]
head(train.0314plant)
nrow(train.0314plant)

# Twenty percentage sample for test
test.0314plant <- grss03to14Spdf_10[setdiff(rownames( grss03to14Spdf_10), rownames(train.0314plant)), ]
nrow(test.0314plant)
head(test.0314plant)

# LRM
grss2plant03to14.binom <- glm(losstoplant03_14RC ~ settwin1k + logd2road + Plant2003mov5In+ slopevarb, family=binomial(link='logit'), data=train.0314plant)
summary(grss2plant03to14.binom)


# Create empty dataframe to store predictions and actuals
library(AUC)
test.0314.rocIn <- data.frame(matrix(vector(), nrow(test.0314plant), 2,
```

```
                    dimnames=list(c(),   c("True.Fate",   "Predictions"))),
stringsAsFactors=F)
test.0314.rocIn$True.Fate <- as.factor(test.0314plant$losstoplant03_14RC)
test.0314.rocIn$Predictions      <-      predict.glm(grss2plant03to14.binom,
newdata=test.0314plant,
                    type='response')

test.0314.roc <- roc(test.0314.rocIn$Predictions, test.0314.rocIn$True.Fate)
test.0314.auc <- auc(test.0314.roc)


save.image(file='F:\\LRMfinalResults\\2003_2014GrasslandLossDTplantations.RData'
)
```

2003 – 2014 Grassland loss due to plantation (15 cells moving window of plantation)

```
setwd('F:/Final/TIFF')
library(raster)
library(rgdal)
library(sp)
library(ROCR)


# LRM inputs for grassland loss to plantculture
grss2plant03to14 <- raster("2014grasslandlossdtplantations.img")
Plant2003mov15In <- raster("F:\\Final\\PLANTATIONDIVIDED.RST")
Sett2003mov1kmIn <- raster("2003settlemets35.tif")
logdist2roadsIn <- raster("Roadlog.tif")
SlopeVar <- raster("Slopevariabilityfloat.tif")
VillageBoundary <- readOGR("F:\\Final\\PalaniHillsVillages.shp")
BeatBoundary <- readOGR("F:\\Final\\KodaikanalBeatDissolve.shp")
```

```
crs(SlopeVar)
crs(logdist2roadsIn)
crs(Sett2003mov1kmIn)
crs(Plant2003mov15In)
crs(grss2plant03to14)

Plant2003mov15In <- projectRaster(Plant2003mov15In, crs=crs(SlopeVar))
Sett2003mov1kmIn <- projectRaster(Sett2003mov1kmIn, crs=crs(SlopeVar))
grss2plant03to14 <- projectRaster(grss2plant03to14, crs=crs(SlopeVar), method="ngb")

# crs(grss2plant03to14) <- crs(logdist2roadsIn)

# Convert grassland change raster to point layer
grss2plant03to14Spdf <- rasterToPoints(grss2plant03to14, spatial=T)

# Change column name and remove zeroes from SpDF
names(grss2plant03to14Spdf) <- c("losstoplant03_14")
grss2plant03to14Spdf_12 <- grss2plant03to14Spdf [grss2plant03to14Spdf@data$losstoplant03_14 != 0, ]

# Extract values from predictor rasters
grss2plant03to14Spdf_12$Plant2003mov15In <- extract(Plant2003mov15In, grss2plant03to14Spdf_12)
grss2plant03to14Spdf_12$logd2road <- extract(logdist2roadsIn, grss2plant03to14Spdf_12)
grss2plant03to14Spdf_12$settwin1k <- extract(Sett2003mov1kmIn, grss2plant03to14Spdf_12)
grss2plant03to14Spdf_12$slopevarb <- extract(SlopeVar, grss2plant03to14Spdf_12)
# grss2plant03to14Spdf_12$villagecd <- as.list(over(grss2plant03to14Spdf_12, VillageBoundary))
```

```
# Recode grassland no-change from 2 to 0
grss2plant03to14Spdf_12$losstoplant03_14RC <- ifelse(grss2plant03to14Spdf_12@data$losstoplant03_14 == 2, 0, grss2plant03to14Spdf_12@data$losstoplant03_14)


# adding xy information, remove NAs from data, remove old column
grss2plant03to14Spdf_12$lat <- grss2plant03to14Spdf_12@coords[,2]
grss2plant03to14Spdf_12$lon <- grss2plant03to14Spdf_12@coords[,1]

grss03to14Spdf_10 <- na.omit(grss2plant03to14Spdf_12@data)
grss03to14Spdf_10 <- grss03to14Spdf_10[,c(2:8)]

# grss03to14Spdf_10VC <- na.omit(grss2plant03to14Spdf_12@data)
# grss03to14Spdf_10VC <- grss03to14Spdf_10VC[,c(2:5, 7:9)]


# Checking for correlations
head( grss03to14Spdf_10)
cor( grss03to14Spdf_10)

#sample

nrow( grss03to14Spdf_10)

(234207 /100)*80

(234207 /100)*20
```

```
# Eighty percentage sample for train

train.0314plant <- grss03to14Spdf_10[sample(1:nrow( grss03to14Spdf_10), 187366,replace=FALSE),]
head(train.0314plant)
nrow(train.0314plant)

# Twenty percentage sample for test
test.0314plant <- grss03to14Spdf_10[setdiff(rownames( grss03to14Spdf_10), rownames(train.0314plant)), ]
nrow(test.0314plant)
head(test.0314plant)

# LRM
grss2plant03to14.binom <- glm(losstoplant03_14RC ~ settwin1k + logd2road + Plant2003mov15In+ slopevarb, family=binomial(link='logit'), data=train.0314plant)
summary(grss2plant03to14.binom)


# Create empty dataframe to store predictions and actuals
library(AUC)
test.0314.rocIn <- data.frame(matrix(vector(), nrow(test.0314plant), 2,
                              dimnames=list(c(), c("True.Fate", "Predictions"))), stringsAsFactors=F)
test.0314.rocIn$True.Fate <- as.factor(test.0314plant$losstoplant03_14RC)
test.0314.rocIn$Predictions <- predict.glm(grss2plant03to14.binom, newdata=test.0314plant,
                                    type='response')

test.0314.roc <- roc(test.0314.rocIn$Predictions, test.0314.rocIn$True.Fate)
test.0314.auc <- auc(test.0314.roc)
```

```
save.image(file='F:\\LRMfinalResults\\2003_2014GrasslandLossDTplantations_15cell
s.RData')
```
